# MLK4 orchestrates macrophage-induced triple-negative breast cancer invasion and ECM remodeling via enhanced paracrine signaling and NF-κB-MMP axis activation

**DOI:** 10.1038/s41419-026-08689-y

**Published:** 2026-04-01

**Authors:** Alicja Mazan-Bury, Dawid Mehlich, Kamila Karpińska, Michał Łaźniewski, Achilleas Moschos, Vi Nguyen-Phuong Truong, Paweł Jańczak, Anna A. Marusiak

**Affiliations:** 1 Laboratory of Molecular OncoSignalling, IMol Polish Academy of Sciences, Warsaw, Poland; 2 Department of Bacteriology and Biocontamination Control, National Institute of Public Health-NIH, Warsaw, Poland

**Keywords:** Cancer microenvironment, Breast cancer

## Abstract

Tumor-associated macrophages (TAMs) are important mediators of triple-negative breast cancer (TNBC) progression, yet the molecular mechanisms driving this process remain incompletely defined. In this study, we identified MLK4, a member of the MAP3K family, as a regulator of TAM-driven oncogenic processes in TNBC. Using a co-culture of TNBC cells with macrophages, we demonstrated that high MLK4 expression in TNBC is essential for macrophage-induced cancer cell proliferation, extracellular matrix (ECM) remodeling, migration, and invasion. Mechanistically, we showed that the cross-talk between TAMs and TNBC cells drives tumor aggressiveness via an MLK4-dependent mechanism by enhancing NF-κB activation and downstream matrix metalloproteinases (MMPs) expression. We also identified the most prominently upregulated factors, including CXCL1 and IL-8, during the co-culture of macrophages and TNBC cells. We further showed that MLK4 expression correlates with increased macrophage infiltration in TNBC patient samples, indicating its potential role in shaping the immunosuppressive tumor microenvironment. Summarizing, our findings uncover a paracrine signaling involving CXCL1 and MLK4-NF-κB-MMPs axis, which mediates the interactions between TAMs and TNBC cells, enhancing proliferation, mesenchymal transition, ECM remodeling and cancer invasion. This work elucidates a new mechanism of macrophage-induced tumor progression and highlights MLK4 as a promising therapeutic target for disrupting cancer cells-macrophage reciprocal communication in TNBC.

## Introduction

The dynamic cross-talk between cancer cells and the tumor microenvironment (TME) plays a fundamental role in breast cancer progression. Increasing evidence shows that the TME promotes tumor growth, angiogenesis, invasion, metastasis, and therapy resistance by providing a protective niche against treatment-induced cytotoxicity [1, 2]. These insights underscore the importance of investigating cancer-TME interactions to uncover mechanisms of disease progression and therapeutic failure.

The TME comprises a diverse array of non-malignant components, including immune cells, fibroblasts, adipocytes, endothelial cells, and the extracellular matrix (ECM), as well as a wide range of soluble mediators [3]. Among the immune cells present in TME, tumor-associated macrophages (TAMs) are especially prominent in triple-negative breast cancer (TNBC), where they can make up to 50% of tumor mass [4]. TAMs originate from monocytes, which, depending on stimuli, differentiate into two main subtypes, M1 and M2 macrophages, each with distinct functional profiles. The pro-inflammatory M1 phenotype is activated by various factors, including IFN-γ (interferon gamma) and LPS (lipopolysaccharide), whereas the immunosuppressive M2 phenotype is primarily activated in response to IL-4 and IL-13 [5]. M2 macrophages, in particular, are closely associated with tumor-promoting conditions in the TME, and a high infiltration of M2-like TAMs to the tumor correlates with poor prognosis and reduced survival in cancer patients [6–8]. However, the M1/M2 classification represents a simplified model, and in physiological conditions, macrophages exhibit a spectrum of activation states rather than a rigid dichotomy. This is particularly evident in TAMs as they demonstrate significant heterogeneity and plasticity in response to the dynamic TME [9, 10]. Numerous studies have highlighted the complex bidirectional interactions between breast cancer cells and TAMs. For instance, TAMs enhance breast cancer cell proliferation, epithelial-to-mesenchymal transition (EMT), angiogenesis, and cytokine production, while also promoting immune evasion through the secretion of suppressive mediators like IL-10, TGF-β, and CCL22, CCL2 and IL-1β [6, 11, 12]. In turn, breast cancer cells actively shape the immune microenvironment by secreting factors such as M-CSF, IL-4, and IL-10 that skew macrophages toward a pro-tumorigenic phenotype [13–15]. These observations highlight the significance of the feedback loops between TAMs and breast cancer cells in driving malignancy and the need to understand their molecular basis for therapeutic development [16]. However, despite extensive research into TAMs' biology and their cross-talk with TNBC cells, the molecular mechanisms of these interactions remain incompletely understood.

Mixed-Lineage Kinase 4 (MLK4/*MAP3K21*) is a serine/threonine kinase of the MAP3K family implicated in regulating cell migration, apoptosis, and proliferation [17]. By activating the downstream signaling pathways, MLK4 contributes to oncogenic processes in various cancer types and has been linked to resistance to targeted therapies [18–21]. Recently, analysis of genomics and transcriptomics data from large cancer patient datasets revealed gene amplification and mRNA upregulation of MLK4 in invasive breast carcinoma at a high frequency of 23% [22, 23]. The most significant upregulation was detected in TNBC patients, reaching over 50%, which was attributed to its oncogenic role in TNBC progression [24]. Furthermore, the role of MLK4 in breast cancer chemoresistance has been investigated, underscoring its impact on clinical treatment efficacy. The combination of chemotherapeutics with MLK4 silencing induces TNBC cells’ apoptosis and enhances DNA damage via impairing ATM phosphorylation and its targets [25]. Additionally, MLK4 has been found to promote cytokine secretion, which may activate DNA damage response pathways, facilitating DNA repair and contributing to chemoresistance in TNBC [25]. Principally, cytokine secretion may also participate in paracrine signaling, mediating cross-talk with components of the TME and carving the tumor-supportive niche. However, the role of MLK4 signaling in tumor-TME interactions and its regulation by specific cytokines in cancer remains unexplored.

Here, we investigated whether MLK4 could serve as a regulator of the interactions between TNBC cells and TAMs. We applied co-culture techniques using macrophages and TNBC cells expressing high MLK4 to explore how this kinase orchestrates the phenotypic response to the microenvironment. We further dissected the paracrine signaling behind these mechanisms and identified downstream effectors of MLK4 that drive cancer progression. Our findings suggest that MLK4 mediates macrophage-driven pro-oncogenic effects, highlighting its potential as a selective therapeutic target in macrophage-rich TNBC.

## Materials and methods

### Cell lines and reagents

SUM149PT and THP-1 cell lines were purchased from BioIVT and DSMZ, respectively. The HCC1806 cell line was a kind gift from the CRUK Manchester Institute, UK. RAW264.7 was a kind gift from IMDiK Polish Academy of Sciences, Poland. SUM149PT cells were cultured in Ham’s F12 medium with 25 mM HEPES supplemented with 5% Fetal Bovine Serum (FBS), 1% penicillin/streptomycin, 1 µg/ml hydrocortisone and 10 µg/ml insulin. HCC1806, THP-1 and RAW264.7 cells were cultured in RPMI-1640 medium supplemented with 10% FBS, 1% penicillin/streptomycin, 2 mM L-glutamine and 1 mM sodium pyruvate. Cell lines were authenticated by short tandem repeat profiling by ATCC Service at the beginning of the research and screened for mycoplasma regularly.

### Generation of THP-1-derived M1 and M2 macrophages

THP-1-derived M1 and M2 macrophages were generated as described in Mazan et al. [26]. Briefly, THP-1 cells were treated with 150 nM PMA for 24 h, followed by a 24-hour rest period with fresh RPMI-1640 medium. Next, for M1 macrophage differentiation, 100 ng/ml of LPS and 20 ng/ml of IFN-γ were added for 18 h. For M2 macrophage differentiation, 20 ng/ml of IL-4 and 20 ng/ml of IL-13 were added for 48 h.

### Generation of human monocyte-derived macrophages (hMDMs)

To generate mature hMDMs, peripheral blood mononuclear cells (PBMCs) were isolated from buffy coats of healthy human donors, provided by the Regional Blood Center (RCKiK, Warsaw), using the density gradient medium Lymphoprep™ (StemCell, #18061) according to the manufacturer’s instructions. Human CD14⁺CD16⁻ monocytes were then isolated from PBMCs with the EasySep™ Human Monocyte Isolation Kit (StemCell, #193559), following the manufacturer’s protocol. The isolated monocytes were cultured in RPMI-1640 medium supplemented with 10% FBS, 1% penicillin/streptomycin, 2 mM L-glutamine, 1 mM sodium pyruvate, and 20 ng/ml human GM-CSF (PeproTech, #300-03-20UG). The culture medium was refreshed every two days. After seven days of treatment with human GM-CSF, mature hMDMs were ready for experiments.

### Colony formation assay

Colony formation assay following co-culture was previously described [26]. Briefly, THP-1-derived M1/M2 macrophages or hMDMs were generated in 0.4 μm pore size Transwell inserts (Corning, #3412). TNBC cells were pre-seeded into 6-well plates, and after attachment to the plate, inserts with macrophages were placed into the wells with TNBC cells. Control conditions included TNBC cells grown without macrophages. After co-culture, TNBC cells were stained with crystal violet. The clonogenic potential was quantified by measuring the absorbance (OD540, Neo Plate Reader, BioTek) after the solubilization of the dye with 10% acetic acid.

### Migration and invasion assays

A co-culture-based migration assay was previously described [26]. Briefly, THP-1-derived M2 macrophages or hMDMs were generated in 24-well plates. TNBC cells were seeded into 8 μm pore size Transwell inserts (Corning, #353097), and the inserts were placed into the wells with macrophages. For the invasion assay, 8 μm pore size Transwell inserts were covered with a layer of Matrigel (Corning, #354480). Control conditions included TNBC cells seeded into 8 μm pore size Transwell inserts and placed into the wells without macrophages. After 24 h, TNBC cells that migrated or invaded through the inserts were stained with crystal violet and pictures were taken. Results were quantified by ImageJ area measurement.

### Statistical analysis

All experiments were performed at least three times. Statistical analyses were carried out with GraphPad Prism 7 software. Data are expressed as the mean ± the standard error of the mean (SEM). *P*-value < 0.05 was considered significant: *p < 0.05, **p < 0.01, ***p < 0.001, ****p < 0.0001. For the experiments where the comparison was performed between more than two groups, the statistical significance was assessed by one-way ANOVA or two-way ANOVA followed by Tukey post-hoc tests. Differences in CIBERSORTx-inferred levels of monocytes and macrophages between groups were assessed using a two-sided Wilcoxon rank-sum test.

For additional methods, please see the Supplementary Information File. Uncropped original western blots are in Supplementary Information File 2.

### Ethics approval and consent to participate

All methods were performed in accordance with the relevant guidelines and regulations. Cell lines used in this study were obtained from commercial sources and did not require ethical approval. Animal procedures were described previously [25] and approved by the Local Ethics Committee at the University of Warsaw (approval no. 1035/2020), and were carried out in accordance with EU Directive 2010/63/EU and Polish legislation (Dz. U. poz. 266, 15 January 2015). This study did not involve human participants.

## Results

### MLK4 plays a critical role in the enhanced proliferation and migration of TNBC cells mediated by pro-tumorigenic macrophages

**Fig. 1:**
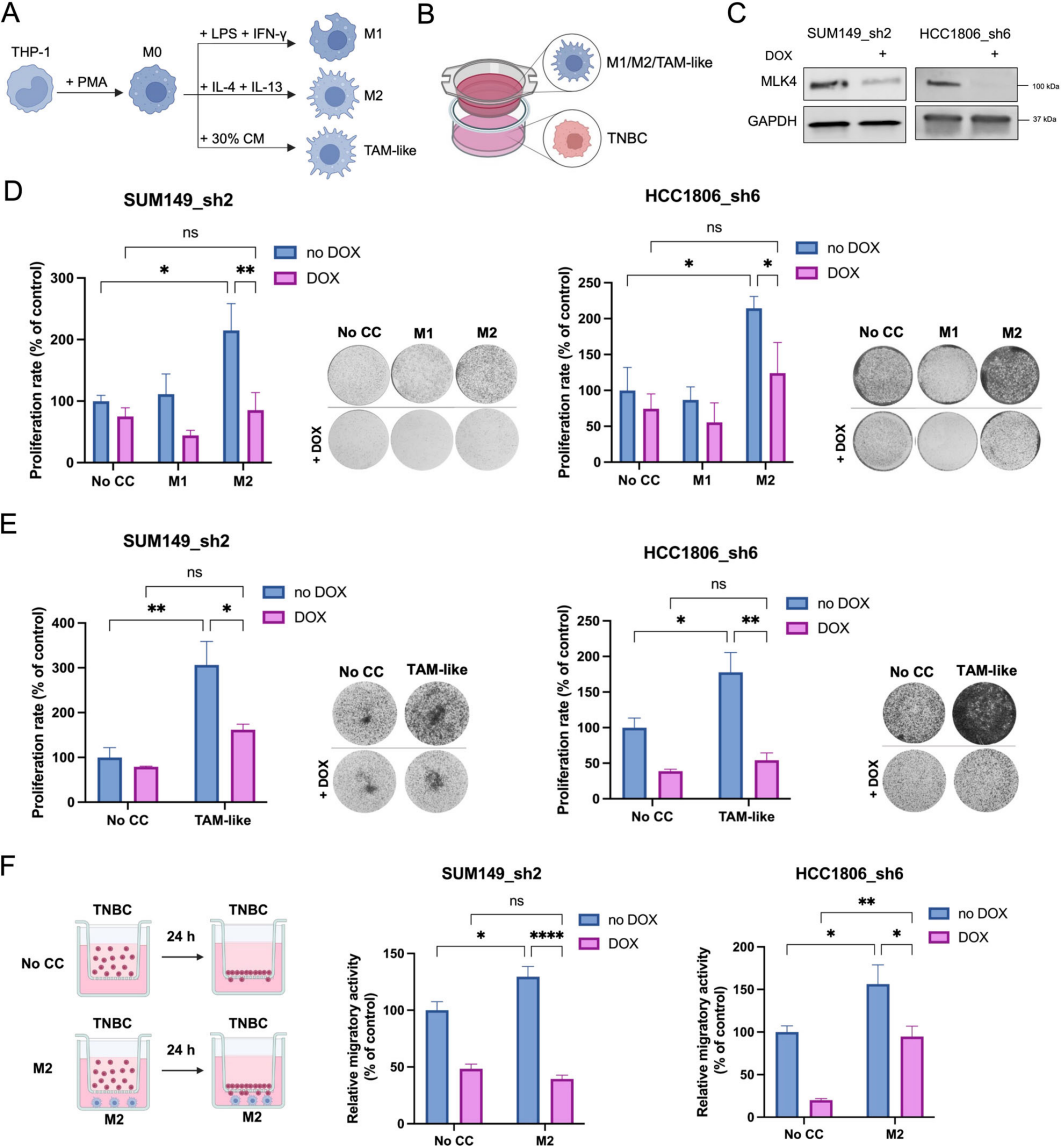
MLK4 knockdown decreases the proliferation and migration of TNBC cells mediated by THP-1-derived M2 macrophages. **A** Schematic illustration of M0, M1, M2 macrophages and TAM-like generation. **B** Schematic illustration of co-culture of macrophages and TNBC cells using 0.4 μm Transwell inserts. **C** Cell lines with doxycycline-inducible MLK4 knockdown (SUM149_sh2 and HCC1806_sh6) were generated from SUM149PT and HCC1806 cells using lentiviral vectors. Silencing of MLK4 was confirmed by immunoblotting. THP-1-derived M1, M2 macrophages (**D**) and TAM-like (**E**) were generated in 0.4 μm Transwell inserts. Briefly, THP-1 cells were treated with 150 nM PMA for 24 h, followed by a 24 h rest period with fresh culture media (**D**, **E**). For M1, cells were incubated with 100 ng/ml LPS and 20 ng/ml IFN-γ for 18 h (**D**). For M2, cells were incubated with 20 ng/ml IL-4, and 20 ng/ml IL-13 for 48 h (**D**). For TAM-like macrophages, cells were incubated for 48 h with 30% conditioned media (CM) collected from the breast cancer cells (**E**). SUM149_sh2 and HCC1806_sh6 were pre-seeded into 6-well plates and treated with doxycycline to induce knockdown of MLK4. After 24 h, inserts with M1, M2 macrophages (**D**) or TAM-like (**E**) were added to the wells with SUM149_sh2 and HCC1806_sh6 for a co-culture period. Control conditions included TNBC cells grown without macrophages (No CC). After 4 days, TNBC cells were stained with crystal violet and pictures were taken. The proliferation rate was quantified by measuring the absorbance (OD540 nm) after solubilization of the dye. Data represent mean results from three independent experiments (error bars ±SEM). Significance was calculated using two-way ANOVA followed by the Tukey multiple comparisons test. **F** Left, schematic illustration of the migration assay for breast cancer cells migrating directly to M2 macrophages. Briefly, THP-1-derived M2 macrophages were generated in 24-well plates as previously described. SUM149_sh2 and HCC1806_sh6 were pre-treated with doxycycline to induce knockdown of MLK4. After 48 h, SUM149_sh2 and HCC1806_sh6 cells were seeded into 8 μm pore size Transwell inserts placed in the wells with M2 macrophages. Control conditions included TNBC cells seeded into 8 μm pore size Transwell inserts placed in the wells without macrophages (No CC). After 24 h, the TNBC cells that migrated were stained with crystal violet and the pictures were taken. Results were quantified by ImageJ. Data represent mean results from three independent experiments (error bars ± SEM). Significance was calculated using two-way ANOVA followed by the Tukey multiple comparisons test.

To assess whether elevated MLK4 expression in TNBC cells is required for interaction with TAMs, we first examined whether macrophages influence breast cancer cell proliferation through MLK4. To this end, we utilized the THP-1 human leukemia monocytic cell line alongside TNBC cell lines. The THP-1 cell line is a well-established model for studying macrophage behavior, as it can be polarized into classical, inflammatory M1 macrophages or alternative, pro-oncogenic M2 macrophages using specific factors (Fig. 1A) [26, 27]. The successful generation of M1 and M2 macrophage phenotypes was confirmed by checking the expression of M1 and M2 markers using two independent techniques: FACS analysis and RT-qPCR (Supplementary Fig. 1A–C). Then, we evaluated the impact of macrophages on the clonogenic potential of breast cancer cells by phenotypic assays using the previously established Transwell co-culture system (Fig. 1B) [26]. We utilized two TNBC cell lines, SUM149PT and HCC1806, with high endogenous MLK4 expression [24, 25]. A doxycycline-inducible MLK4 knockdown system, using two different lentiviral shRNA vectors, was previously developed in our laboratory (Fig. 1C and Supplementary Fig. 2A) [24, 25]. We co-cultured M1 or M2 macrophages together with breast cancer cells (with or without doxycycline-induced MLK4 knockdown). Our data indicated that the proliferation of TNBC cells was enhanced by the presence of M2 macrophages, in agreement with prior reports [28, 29], while MLK4 silencing significantly attenuated this effect (Fig. 1D and Supplementary Fig. 2B). The knockdown of MLK4 alone reduced TNBC cell proliferation, consistent with our previous findings [24]. Importantly, MLK4 silencing resulted in an even more pronounced reduction in proliferation, abolishing the macrophage-induced proliferative effect observed in control cells (Fig. 1D). This indicates that MLK4 is required for M2 macrophages to stimulate TNBC cell proliferation. In contrast, co-culture with pro-inflammatory classical M1 macrophages did not increase TNBC cell proliferation, which is consistent with the literature (Fig. 1D) [30].

To exclude the possibility that the observed effects were due to doxycycline treatment itself, we treated the parental cell lines, SUM149PT and HCC1806, with doxycycline and conducted co-culture experiments with THP-1-derived M2 macrophages. Doxycycline treatment did not affect the proliferation of SUM149PT and HCC1806 parental cell lines nor their response to macrophages (Supplementary Fig. 3). These results demonstrate that doxycycline neither affected the growth of TNBC cells nor influenced the macrophage-induced increase in cancer cell proliferation (Supplementary Fig. 3). Moreover, this confirmed that the effects observed in doxycycline-inducible MLK4-knockdown cell lines were attributable to MLK4 silencing.

TAMs in the TME often exhibit a hybrid phenotype, combining features of both pro-inflammatory (M1-like) and immunosuppressive (M2-like) states [31, 32]. This plasticity enables them to adapt to tumor progression by supporting immune modulation, matrix remodeling, and angiogenesis [33–36]. With this in mind, we generated THP-1-derived TAM-like macrophages by culturing them with conditioned media (CM) from breast cancer cells, rather than adding M1- or M2-differentiating factors (Fig. 1A). We first confirmed the successful generation of TAM-like macrophages using FACS analysis and RT-qPCR (Supplementary Fig. 1A–C). TAM-like macrophages generated using CM strongly expressed M2 markers while also exhibiting some M1 markers (Supplementary Fig. 1C). We further demonstrated that the presence of THP-1-derived TAM-like macrophages enhanced the proliferation of TNBC cells, whereas MLK4 knockdown significantly reduced this effect (Fig. 1E and Supplementary Fig. 2C).

Since macrophages promote breast cancer cell migration, a key aspect of tumor aggressiveness, we investigated whether this pro-migratory effect requires MLK4 signaling in cancer cells. We assessed the migratory properties of TNBC cells using a Transwell migration assay (Fig. 1F). We observed that the migration of SUM149_sh2 and HCC1806_sh6 cells was significantly increased in the presence of THP-1-derived M2 macrophages as in previous reports [37, 38], while this effect was abrogated by MLK4 silencing in both TNBC cell lines (Fig. 1F). These observations were confirmed using the wound healing assay as an alternative method to assess cancer cell migration. As in the Transwell assay, M2 macrophages increased TNBC cell migration, and this was reduced upon MLK4 silencing (Supplementary Fig. 4). These findings demonstrate that M2 macrophages enhance the migratory features of breast cancer cells in an MLK4-dependent manner. Combined, our findings indicate that high MLK4 expression in TNBC cells is required for enabling the pro-tumorigenic activity of macrophages.

**Fig. 2:**
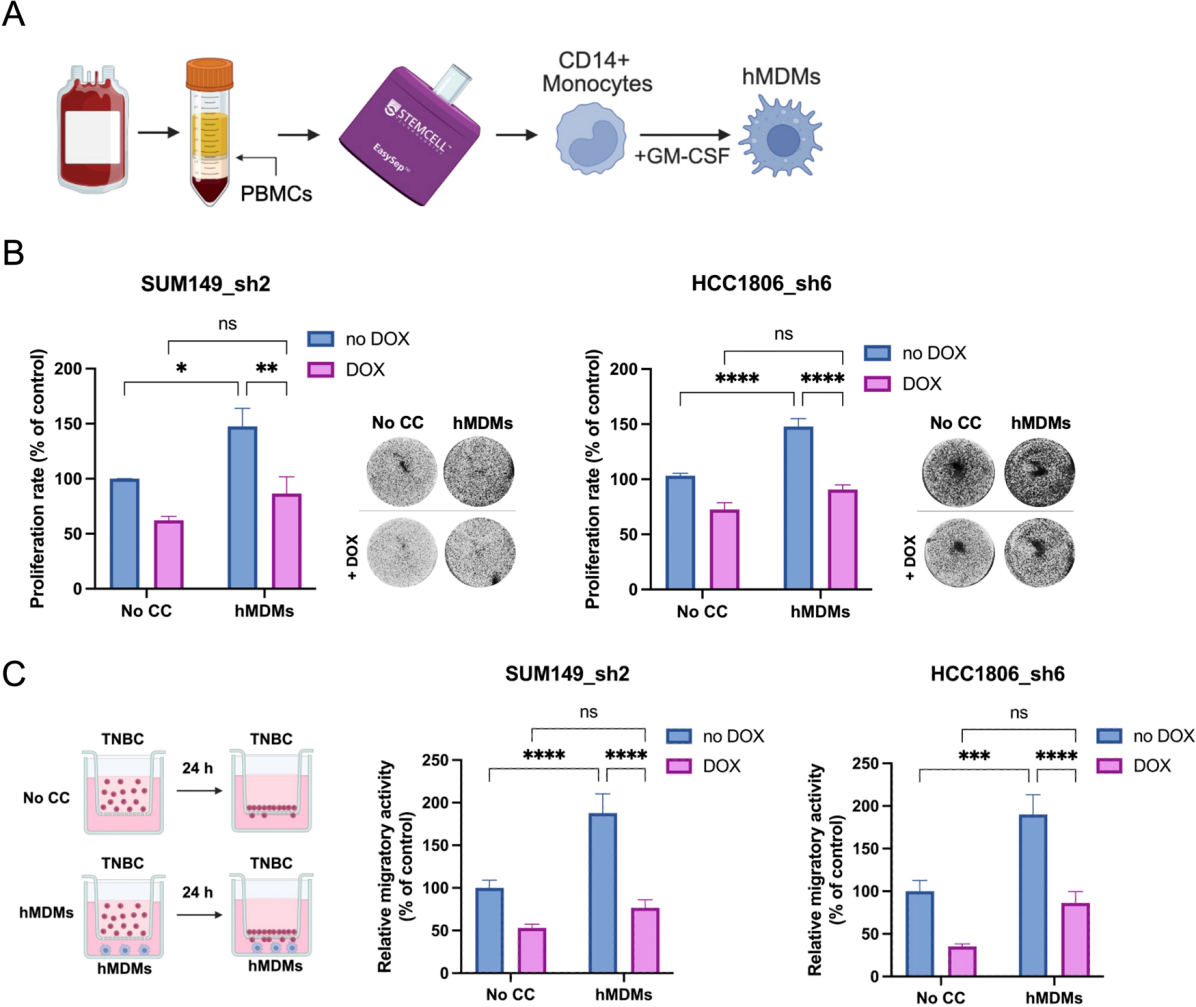
MLK4 knockdown decreases the proliferation and migration of TNBC cells mediated by hMDMs. **A** Schematic illustration of hMDMs generation. **B** hMDMs were generated in 0.4 μm Transwell inserts. SUM149_sh2 and HCC1806_sh6 were pre-seeded into 6-well plates and treated with doxycycline to induce knockdown of MLK4. After 24 h, inserts with hMDMs were added to the wells with SUM149_sh2 and HCC1806_sh6 for a co-culture period. Control conditions included TNBC cells grown without macrophages (No CC). After 4 days, TNBC cells were stained with crystal violet and pictures were taken. The proliferation rate was quantified by measuring the absorbance (OD540 nm) after solubilization of the dye. Data represent mean results from at least three independent experiments (error bars ± SEM). Significance was calculated using two-way ANOVA followed by the Tukey multiple comparisons test. **C** Left, schematic illustration of the migration assay for breast cancer cells migrating directly to hMDMs. hMDMs were generated in 24-well plates. SUM149_sh2 and HCC1806_sh6 were pre-treated with doxycycline to induce knockdown of MLK4. After 48 h, SUM149_sh2 and HCC1806_sh6 cells were seeded into 8 μm pore size Transwell inserts placed in the wells with hMDMs. Control conditions included TNBC cells seeded into 8 μm pore size Transwell inserts and placed into the wells without macrophages (No CC). After 24 h, the TNBC cells that migrated were stained with crystal violet and the pictures were taken. Results were quantified by ImageJ. Data represent mean results from three independent experiments (error bars ± SEM). Significance was calculated using two-way ANOVA followed by the Tukey multiple comparisons test.

THP-1 cells may not fully replicate the diversity of primary human macrophages; it is crucial to validate the findings using an alternative macrophage model. We employed human monocyte-derived macrophages (hMDMs), isolated from the peripheral blood mononuclear cell (PBMC) fraction of healthy donors, and differentiated from monocytes into macrophages through culture with huGM-CSF (Fig. 2A) [39]. This model is more physiologically accurate and biologically relevant, exposing the plasticity of primary macrophages compared to immortalized cell lines like THP-1. We first confirmed the efficiency of monocyte isolation by FACS analysis of CD14 levels (Supplementary Fig. 5A), followed by analysis of macrophage markers after huGM-CSF treatment, which showed that the majority of differentiated hMDMs were CD206-positive and CD80-negative (Supplementary Fig. 5B, C). Additional RT-qPCR analysis confirmed M2-like phenotype of hMDMs (Supplementary Fig. 6). Next, we performed colony formation and migration assays using the TNBC cell lines, SUM149_sh2 and HCC1806_sh6, after co-culture with hMDMs. Our results validated that MLK4 is essential for the increased proliferation rate (Fig. 2B) and migration of breast cancer cells driven by hMDMs (Fig. 2C), consistent with data obtained using the THP-1 macrophage model (Fig. 1D–F).

### MLK4 is essential for macrophage-induced gene expression changes associated with extracellular matrix remodeling, cell adhesion, and invasion

**Fig. 3:**
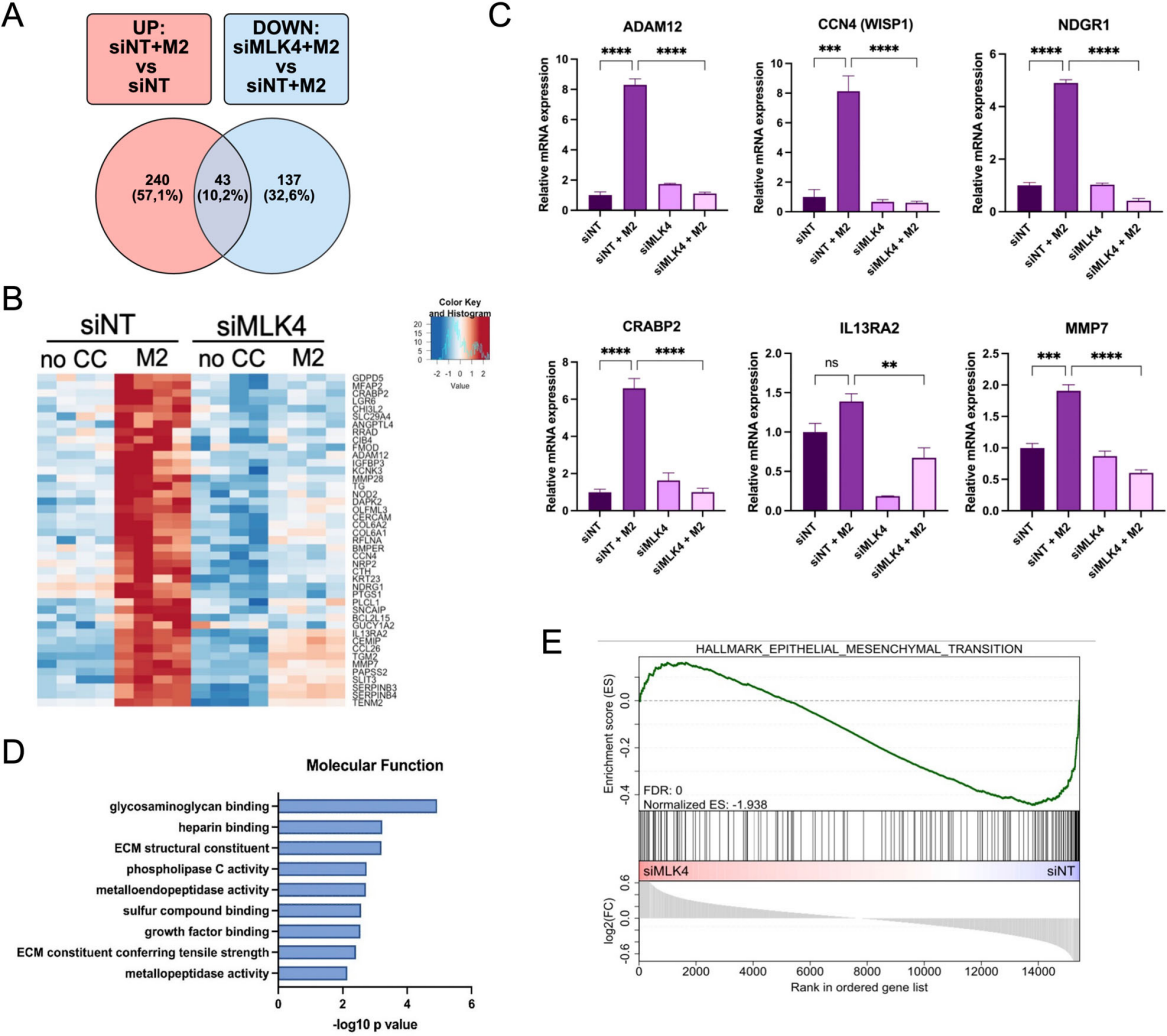
MLK4 loss compromises transcriptional changes in TNBC cells induced by the presence of macrophages. **A** Venn diagram presenting the overlapping genes that are upregulated after co-culture with M2 macrophages and significantly downregulated in MLK4-depleted cells compared to control cells after the co-culture. **B** Heatmap depicting the transcriptome-wide effects of MLK4 depletion and co-culture with M2 macrophages. Shown are significantly [pvalue adj. < 0.01] upregulated [log2 FC > 0.75, red] or downregulated [log2 FC < 0.75], blue] genes. **C** SUM149PT cells were transfected with non-targeting siRNA (siNT) or MLK4-targeting siRNA (siMLK4). Following the transfection, cells were co-cultured with THP-1-derived M2 macrophages for 48 h. The RNA was isolated, and relative expression of several genes was analyzed by RT-qPCR (error bars ± SEM). Significance was calculated using one-way ANOVA followed by the Tukey multiple comparisons test. **D** GO enrichment analysis in MLK4-depleted vs control cells co-cultured with M2 macrophages. **E** GSEA in MLK4-depleted vs control cells co-cultured with M2 macrophages.

To gain insights into the molecular mechanisms involved in macrophage-induced breast cancer cell proliferation and the role of MLK4 signaling, we performed mRNA-seq. We analyzed SUM149PT cells with and without MLK4 silencing, under co-culture conditions with THP-1-derived M2 macrophages. To prevent gene expression changes associated with doxycycline-inducible knock-down, we silenced MLK4 using transient transfection, which effectively reduced MLK4 transcript levels (Supplementary Fig. 7A). PCA analysis confirmed the high reproducibility of our mRNA-seq data (Supplementary Fig. 7B). Our analysis focused on genes that were upregulated in response to co-culture with macrophages (UP: siNT+M2 vs. siNT) but downregulated following MLK4 silencing in co-culture conditions (DOWN: siMLK4 + M2 vs. siNT+M2) (Fig. 3A). MLK4 depletion caused a pronounced alteration in the transcriptome of TNBC cells induced by M2 macrophages, identifying a distinct set of 43 genes (Fig. 3B). Among these, we identified several secreted factors and metalloproteinases, including MMP28, MMP7 and ADAM12. To validate the mRNA-seq results, we analyzed the expression of selected genes by RT-qPCR in MLK4-depleted SUM149PT and control cells with or without M2 macrophages. Consistent with our mRNA-seq data, the expression of ADAM12, CCN4 (WISP1), CRABP1, MMP7, NDGR1, and IL13RA2 was induced by M2 macrophages but decreased in MLK4-silenced, co-cultured TNBC cells (Fig. 3C).

We then performed functional enrichment analysis of the 43 genes downregulated following MLK4 depletion in cells co-cultured with M2 macrophages. Gene ontology (GO) term analysis revealed significant enrichment of the key processes involved in cancer cell invasion and ECM remodeling (Fig. 3D). For instance, glycosaminoglycan and heparin binding facilitate interactions with ECM components, influencing adhesion, signaling, and motility [40, 41]. Extracellular matrix structural constituent functions, including those conferring tensile strength, involve proteins such as collagens that maintain ECM integrity and modulate stiffness, impacting cell migration [42]. Metalloendopeptidase and metallopeptidase activities promote ECM remodeling by degrading structural components, creating migration pathways, and releasing bioactive fragments [42]. Furthermore, Gene Set Enrichment Analysis (GSEA) identified the EMT Hallmark as one of the top negatively enriched gene sets in co-culture conditions after MLK4 silencing (Fig. 3E). These findings suggested that MLK4 signaling plays an essential role in regulating ECM reorganization, EMT, and cancer cell invasion in response to microenvironmental cues.

### MLK4 regulates macrophage-driven extracellular matrix remodeling and cell invasion

**Fig. 4:**
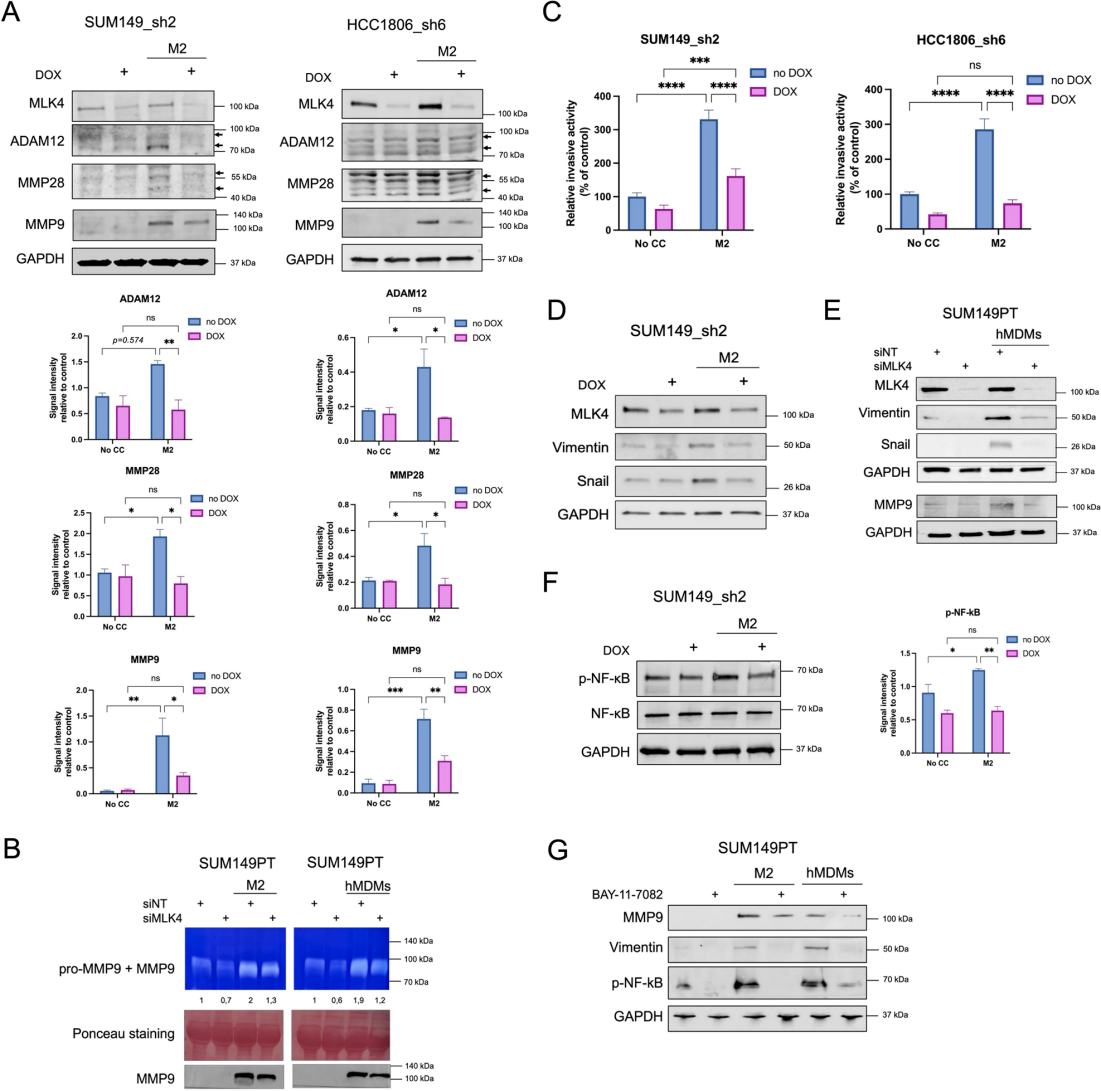
MLK4 knockdown impairs macrophage-driven ECM remodeling and TNBC invasive phenotype. **A** THP-1-derived M2 macrophages were generated in 0.4 μm Transwell inserts. Briefly, THP-1 cells were treated with 150 nM PMA for 24 h, followed by a 24 h rest period with fresh culture media, then cells were incubated with 20 ng/ml IL-4 and 20 ng/ml IL-13 for 48 h. SUM149_sh2 and HCC1806_sh6 were pre-seeded into 6-well plates and treated with doxycycline to induce knockdown of MLK4. After 24 h, inserts with M2 macrophages were added to the wells with SUM149_sh2 and HCC1806_sh6 for a co-culture period. Control conditions included TNBC cells grown without macrophages. After 48 h, cells were lysed, and whole cell lysates were analyzed by immunoblotting. Quantification analysis of ADAM12, MMP28 and MMP9 immunoblots was performed using ImageJ. Data represent mean results from three independent experiments (error bars ±SEM). Significance was calculated using two-way ANOVA followed by the Tukey multiple comparisons test. **B** THP-1-derived M2 macrophages and hMDMs were generated in 0.4 μm Transwell inserts as previously described. SUM149PT were pre-seeded into 6-well plates and transfected with non-targeting siRNA (siNT) or MLK4-targeting siRNA (siMLK4). After 24 h, SUM149PT were co-cultured with M2 macrophages or hMDMs for another 48 h. After discarding the Transwell inserts, the media was changed for fresh, and the TNBC cells were cultured for an additional 24 h. The supernatants were then collected and subjected to gelatin zymography assay. A representative image from three independent experiments is shown. Band intensity was quantified using ImageJ for MMP9. Ponceau S staining served as a loading control. Additionally, the supernatants from TNBC cells were analyzed by immunoblotting for MMP9. **C** THP-1-derived M2 macrophages were generated in 24-well plates as previously described. SUM149_sh2 and HCC1806_sh6 were pre-treated with doxycycline to induce knockdown of MLK4. After 48 h, SUM149_sh2 and HCC1806_sh6 cells were seeded into 8 μm pore size Transwell inserts covered with Matrigel, and placed in the wells with M2 macrophages. Control conditions included TNBC cells seeded into 8 μm pore size Transwell inserts and placed in the wells without macrophages (No CC). After 24 h, TNBC cells that invaded through the inserts were stained with crystal violet and the pictures were taken. Results were quantified by ImageJ. Data represent mean results from three independent experiments (error bars ±SEM). Significance was calculated using two-way ANOVA followed by the Tukey multiple comparisons test. **D** M2 macrophages were generated in 0.4 μm Transwell inserts. SUM149_sh2 were pre-seeded into 6-well plates and treated with doxycycline to induce knockdown of MLK4. After 24 h, inserts with M2 macrophages were added to SUM149_sh2 for co-culture. Control conditions included TNBC cells grown without macrophages. After 48 h, TNBC cells were lysed, and whole cell lysates were analyzed by immunoblotting. **E** hMDMs were generated in 0.4 μm Transwell inserts as previously. SUM149PT were pre-seeded into 6-well plates and transfected with non-targeting siRNA (siNT) or MLK4-targeting siRNA (siMLK4). After 24 h, inserts with hMDMs were added to SUM149PT cells. Control conditions included TNBC cells grown without hMDMs. After 48 h, TNBC cells were lysed, and whole cell lysates were analyzed by immunoblotting. **F** THP-1-derived M2 macrophages were generated in 0.4 μm Transwell inserts as previously described. SUM149_sh2 were pre-seeded into 6-well plates and treated with doxycycline to induce knockdown of MLK4. After 24 h, inserts with M2 macrophages were added to SUM149_sh2 for co-culture. Control conditions included TNBC cells grown without macrophages. After 48 h, TNBC cells were lysed, and whole cell lysates were analyzed by immunoblotting. Quantification analysis of p-NF-κB immunoblot was performed using ImageJ. Data represent mean results from three independent experiments (error bars ± SEM). Significance was calculated using two-way ANOVA followed by the Tukey multiple comparisons test. **G** M2 macrophages and hMDMs were generated in 0.4 μm Transwell inserts as previously. SUM149PT cells were pre-seeded into 6-well plates. After 24 h, inserts with M2 macrophages or hMDMs were added to SUM149PT cells for co-culture, with or without 10 μM BAY-11-7082 inhibitor. Control conditions included SUM149PT cells grown without macrophages. After 48 h, TNBC cells were lysed, and whole cell lysates were analyzed by immunoblotting.

Since our mRNA-seq analysis revealed that MLK4 played a crucial role in macrophage-induced transcriptional activation of genes involved in ECM remodeling, EMT, and cell invasion, we aimed to validate these findings further. First, we assessed whether MLK4 knockdown in TNBC cells affected the expression of proteins associated with ECM regulation, including ADAM12, MMP28 and additionally MMP9, due to its well-established role in tumor angiogenesis, invasion and metastatic progression [43, 44]. Our results confirmed that the expression of these metalloproteinases in TNBC cells was significantly upregulated in the presence of M2 macrophages but reduced after MLK4 knockdown (Fig.4A and Supplementary Fig.8). Following this, we examined the functional activity of MMP9 using a gelatin zymography assay. SUM149PT cells with or without MLK4 knockdown were co-cultured for 48 h with either THP-1-derived M2 macrophages or hMDMs (Supplementary Fig. 9). Since macrophages secrete high levels of MMPs, after the co-culture period, we separated TNBC cells from macrophages, changed to the fresh culture media, and after 24 h, collected the conditioned supernatants to measure the activity of MMPs secreted only by TNBC cells. Zymography assays revealed that the co-culture with macrophages strongly enhanced TNBC MMP9 activity, and MLK4 silencing prevented such an increase, supporting MLK4’s role in regulating macrophage-induced proteolytic remodeling of the ECM by TNBC cells (Fig. 4B).

We next investigated whether the proteolytic remodeling described above contributes functionally to the invasive behavior of TNBC cells. Invasion assays revealed that co-culture of TNBC cells, SUM149_sh2 and HCC1806_sh6, with THP-1-derived M2 macrophages markedly enhanced TNBC invasion through Matrigel. Importantly, this pro-invasive effect was significantly reduced upon MLK4 depletion in TNBC cells (Fig. 4C). These findings support that MLK4 is an essential mediator of ECM remodeling that facilitates TNBC cell invasion.

To validate whether TAMs induced EMT changes in an MLK4-dependent manner, we examined EMT marker expression in TNBC cells co-cultured with THP-1-derived M2 macrophages. Co-culture with macrophages upregulated EMT markers in TNBC cells (Vimentin and Snail), and this effect was abolished in MLK4-depleted TNBC cells (Fig. 4D). Similarly to the THP-1-derived macrophage model, MMP9 and EMT marker expression in SUM149_sh2 cells increased after co-culture with hMDMs, and this increase was reduced upon MLK4 silencing (Fig. 4E).

Prior studies showed that elevated MLK4 expression in TNBC and glioma cells activates the NF-κB pathway, promoting a mesenchymal phenotype switch and aggressive tumor growth [21, 24]. Consistent with these findings, we asked whether MLK4 modulated NF-κB pathway activation in TNBC cells under co-culture with macrophages. Indeed, our data revealed that the presence of macrophages increased the activation of the NF-κB pathway in TNBC cells, but MLK4 silencing attenuated this effect (Fig. 4F and Supplementary Fig. 10A, B), suggesting that macrophage-induced NF-κB activation in TNBC requires MLK4. NF-κB is known to directly regulate the transcription of EMT-related genes and several MMP genes, including MMP9 [45]. Therefore, we tested if pharmacological inhibition of NF-κB signaling using the small-molecule inhibitor, BAY-11-7082, affected the expression of Vimentin and MMP9 during co-culture with macrophages. NF-κB pathway inhibition reduced the expression of Vimentin and MMP9, which was induced by the co-culture in both macrophage models, THP-1-driven M2 macrophages and hMDMs (Fig. 4G). NF-κB inhibition recapitulated the effects of MLK4 knockdown, confirming the importance of an MLK4-NF-κB axis in the context of macrophage-driven cancer progression. Collectively, our data support a model in which MLK4 acts upstream of the NF-κB pathway, mediating macrophage-induced EMT changes and MMPs expression, leading to ECM remodeling and increased invasion of TNBC cells.

### MLK4 mediates paracrine signaling during TNBC-macrophage interactions

**Fig. 5:**
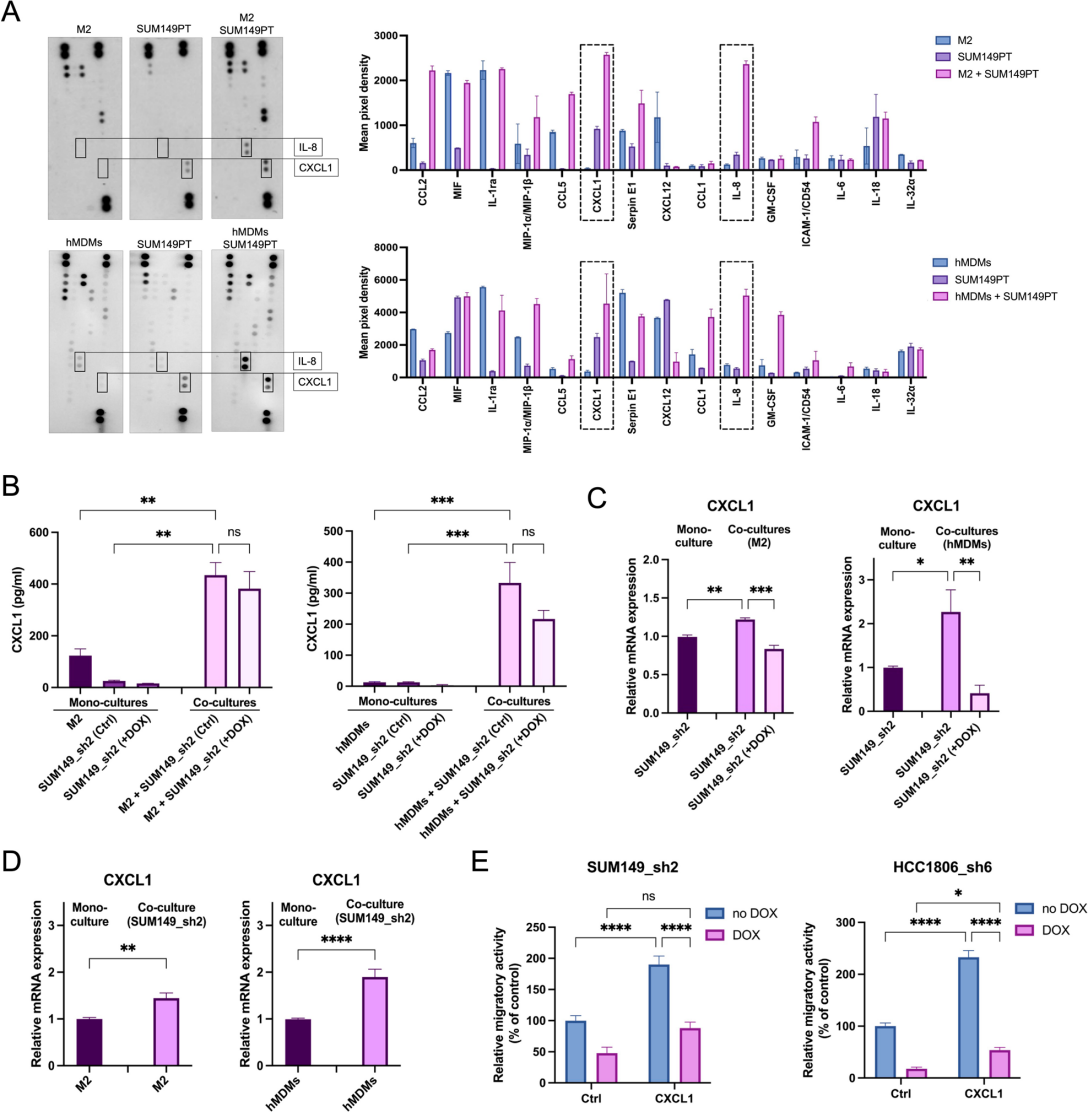
CXCL1 secretion is enhanced by TNBC-macrophage interactions and promotes TNBC migration in an MLK4-dependent manner. **A** THP-1-derived M2 macrophage and hMDMs were generated in 0.4 μm Transwell inserts as previously. SUM149PT cells were pre-seeded in 6-well plates and co-cultured with the generated M2 macrophages or hMDMs. Control conditions included SUM149PT and macrophage monocultures. After 24 h, supernatants from mono-cultures and co-cultures were collected and subjected to Cytokine Array. Quantification analysis of the arrays was performed using ImageJ. **B**–**D** THP-1-derived M2 macrophages and hMDMs were generated in 0.4 μm Transwell inserts. SUM149_sh2 were pre-seeded into 6-well plates and treated with doxycycline to induce knockdown of MLK4. After 24 h, inserts with M2 macrophages or hMDMs were added to SUM149_sh2 for co-culture. Control conditions included SUM149_sh2 and macrophage mono-cultures. After 24 h, supernatants from mono-cultures and co-cultures were collected and subjected to ELISA assay (error bars ± SEM) (**B**), while the cells from mono-cultures and co-cultures were lysed, RNA was isolated, and relative expression of several genes was analyzed by RT-qPCR (error bars ± SEM) (**C**, **D**). Significance was calculated using one-way ANOVA (**B**–**D**) followed by the Tukey multiple comparisons test. **E** SUM149_sh2 and HCC1806_sh6 were pre-seeded in 6 well plate and treated with doxycycline to induce knock-down of MLK4. After 48 h, SUM149_sh2 and HCC1806_sh6 cells were trypsinized and seeded into 8 μm pore size Transwell inserts placed in the wells with or without 40 ng/ml huCXCL1. After 24 h, the TNBC cells that migrated were stained with crystal violet and the pictures were taken. Results were quantified by ImageJ. Data represent mean results from three independent experiments (error bars ± SEM). Significance was calculated using two-way ANOVA followed by the Tukey multiple comparisons test.

To investigate the mechanisms underlying MLK4-dependent cross-talk between breast cancer cells and TAM-like macrophages, we sought to identify the paracrine signaling factors involved. Specifically, we characterized the cytokine milieu in co-cultures of macrophages and breast cancer cells with differing MLK4 expression levels. The Human Cytokine Array, which enables the simultaneous detection of 36 cytokines, chemokines, and acute-phase proteins in a single sample, was employed for this purpose. Supernatants from mono-cultures and co-cultures of SUM149PT with THP-1-derived M2 or hMDMs were collected and analyzed (Fig. 5A and Supplementary Fig. 11A). The secretion of several cytokines was specific only for M2 macrophages or hMDMs, or only for SUM149PT cells, and not changed upon the co-culture. However, among the detected factors, CXCL1 and IL-8 emerged as the most prominently elevated factors during co-culture of SUM149PT cells with both models of macrophages, M2 and hMDMs (Fig. 5A). To validate the findings obtained from Cytokine Arrays, the concentrations of CXCL1 and IL-8 were measured by ELISA. We tested supernatants from SUM149_sh2, without and with MLK4 knockdown, and both macrophage models (mono-culture and co-culture conditions). The results were consistent with the arrays, demonstrating that co-culture synergistically increased CXCL1 and IL-8 secretion compared to mono-cultures of SUM149_sh2 cells or macrophages alone (Fig. 5B and Supplementary Fig. 11B). In co-culture conditions, secretion of CXCL1 and IL-8 was modestly reduced in MLK4-depleted TNBC cells compared to controls (Fig. 5B and Supplementary Fig. 11B). Although the decrease was not statistically significant, this trend may be attributed to the fact that macrophages also contributed to the overall CXCL1 and IL-8 secretion in co-culture conditions (Fig. 5B and Supplementary Fig. 11B). To determine which cell type, macrophages or TNBC cells, contributed most to the production of secreted factors, we focused on one of them, CXCL1, and we assessed its expression by RT-qPCR in both cell types (mono-cultures and co-cultures conditions). We found that both TNBC cells and macrophages exhibited increased CXCL1 expression following co-culture, indicating that their mutual interactions may drive this upregulation (Fig. 5C, D). Additionally, MLK4 knockdown in SUM149_sh2 cells significantly reduced CXCL1 expression following co-culture (Fig. 5C). Collectively, these results confirm that co-culture enhances CXCL1 expression in both cell types, leading to synergistically increased secretion, and that CXCL1 paracrine signaling in TNBC cells is regulated by MLK4.

Finally, we investigated whether the addition of CXCL1 instead of co-culture with macrophages could mimic the phenotypic effects observed during the migration assay. SUM149_sh2 and HCC1806_sh6 cells were subjected to a migration assay with or without CXCL1 as a chemoattractant (Fig. 5E). Both cell lines showed significantly enhanced migratory activity in response to CXCL1, while MLK4 depletion in TNBC cells abolished this effect (Fig. 5E). In summary, our findings demonstrate that interactions between cancer cells and TAMs enhance CXCL1 secretion into the TME and promote cancer cell migration in MLK4-dependent manner. Therefore, MLK4, upregulated in TNBC, emerges as a key mediator of both autocrine and paracrine signaling loops that promote tumor progression.

### High MLK4 expression correlates with increased macrophage infiltration in TNBC tumors

**Fig. 6:**
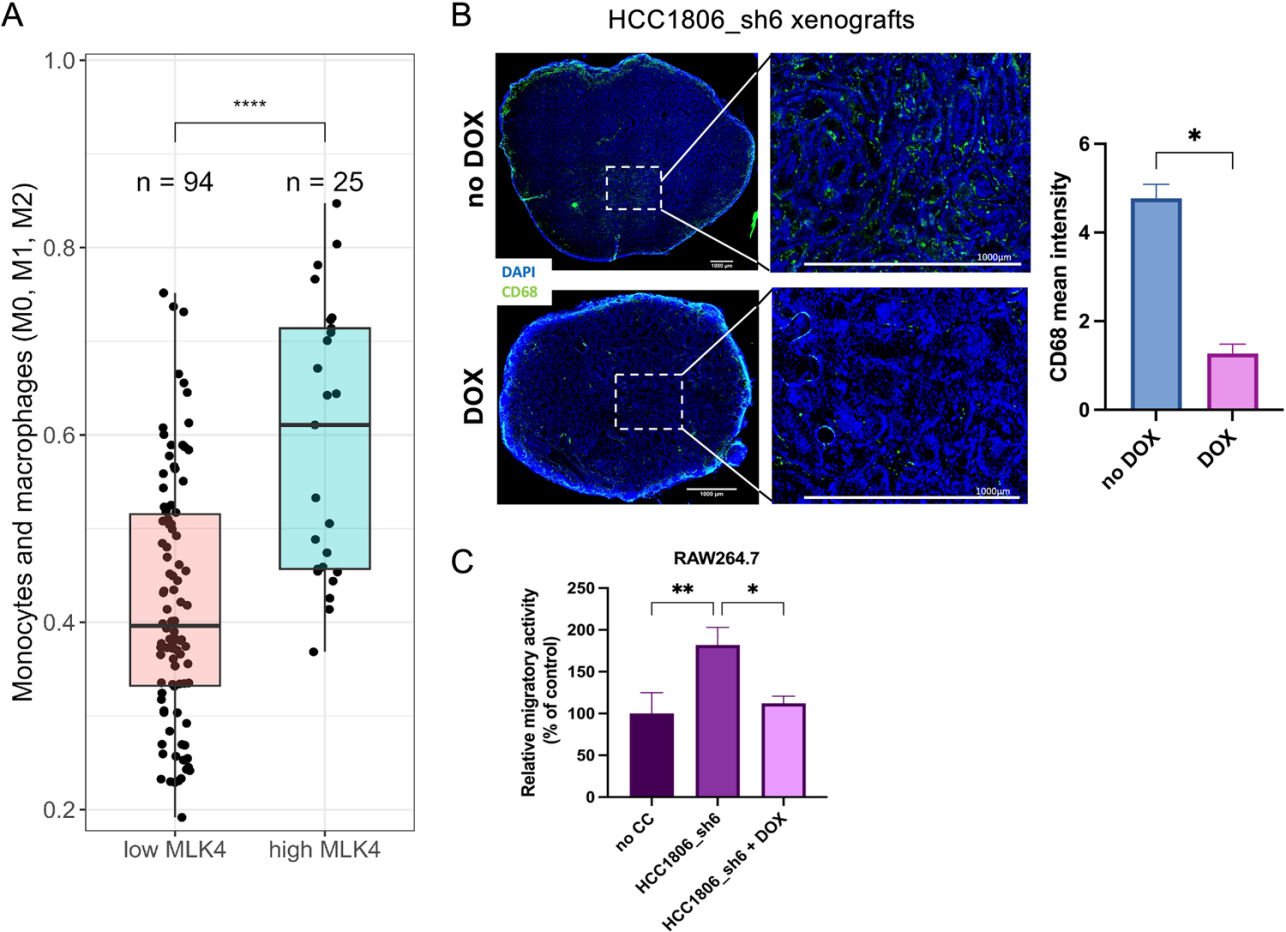
High MLK4 expression correlates with increased macrophage infiltration in TNBC. **A** TNBC tumors were split into two groups based on log₂(TPM) values for the *MLK4* gene. Samples with expression greater than 4 were considered “high MLK4,” and those with expression ≤ 4 were classified as “low MLK4.” This resulted in groups of 25 and 94 samples, respectively. Differences in CIBERSORTx-inferred levels of monocytes and macrophages between groups were assessed using a two-sided Wilcoxon rank-sum test. **B** Immunofluorescent staining of the mouse xenograft samples infiltrated by macrophages. HCC1806_sh6 cells were injected into mammary fat pads of RAG2^–/–^ mice. Doxycycline was administered one day after the injection to induce MLK4 knockdown. Tumors were harvested after 26 days and stained for macrophage marker, CD68 (green) and DAPI (blue). Scale bar 1000 μm. Representative pictures are shown. Quantification of mean CD68 intensity measured in the center of four tumors using ImageJ. Significance was calculated using a t-test (error bars ± SEM). **C** HCC1806_sh6 cells were seeded into 24-well plates and treated with doxycycline to induce MLK4 knockdown. RAW264.7 cells were seeded into 8 μm pore size Transwell inserts and placed in the wells with HCC1806_sh6. Control conditions included RAW264.7 cells seeded into 8 μm pore size Transwell inserts and placed into the wells without TNBC cells (no CC). After 24 h, the RAW264.7 cells that migrated were stained with crystal violet and the pictures were taken. Results were quantified by ImageJ. Data represent mean results from three independent experiments (error bars ± SEM). Significance was calculated using two-way ANOVA followed by the Tukey multiple comparisons test.

To assess the clinical relevance of our finding, we investigated whether high MLK4 expression correlated with increased intra-tumoral macrophage density in TNBC. We previously showed high expression of MLK4 predominantly in TNBC patients’ samples [24]. Based on literature reports indicating that TNBC exhibits prominent immune infiltration, including TAMs [7], we hypothesized that elevated MLK4 expression in TNBC may be linked to an increased presence of tumor-infiltrating immune cells. To explore the clinical relevance of this link, we used CIBERSORTx, an in-silico tool for quantifying immune cells in mRNA-seq or microarray data. We analyzed TCGA data from TNBC patients and observed elevated monocyte and macrophage levels in samples with high MLK4 expression (Fig. 6A).

To further corroborate this correlation, we analyzed tumors from a previously published in vivo xenograft-based experiment conducted by our group, where HCC1806_sh6 cells were injected into RAG2^–/–^ mice, which are immunodeficient for T and B cells but retain functional macrophages [25, 46]. We collected HCC1806_sh6 xenograft tumors from the control group (no MLK4 silencing) and the group where MLK4 silencing was induced by doxycycline administration (Fig. 6B). Tumor samples were stained with the macrophage marker CD68 to investigate the correlation between MLK4 expression levels and macrophage infiltration. Our data demonstrated that CD68 staining intensity was markedly reduced in MLK4-depleted xenograft tumors (Fig. 6B), suggesting that high MLK4 expression in TNBC is associated with increased macrophage infiltration, consistent with the findings from CIBERSORTx.

Given that the xenograft experiment utilized the human TNBC cell line HCC1806 and staining was performed for mouse CD68+ macrophages, we sought to confirm whether mouse macrophages could respond to factors secreted by human cells. To validate this, we used the mouse macrophage cell line RAW264.7 in a migration assay towards HCC1806_sh6 cells. The results showed that RAW264.7 cells exhibited increased migratory activity in response to TNBC cells, while MLK4 depletion in TNBC cells significantly diminished this effect (Fig. 6C). Additionally, we assessed whether human macrophages displayed similar migratory behavior in response to TNBC cells with differing MLK4 expression levels. THP-1-derived macrophages were subjected to a migration assay in the presence or absence of TNBC cells (SUM149_sh2 and HCC1806_sh6). Similar to previous results (Fig. 6C), we observed enhanced macrophage migration in response to TNBC cells, which was reduced upon MLK4 depletion (Supplementary Fig. 12A, B). Our findings demonstrate that high MLK4 expression in TNBC cells enhances macrophage migration in in vitro and in vivo models, further supporting the role of MLK4 in shaping the tumor immune microenvironment.

## Discussion

**Fig. 7:**
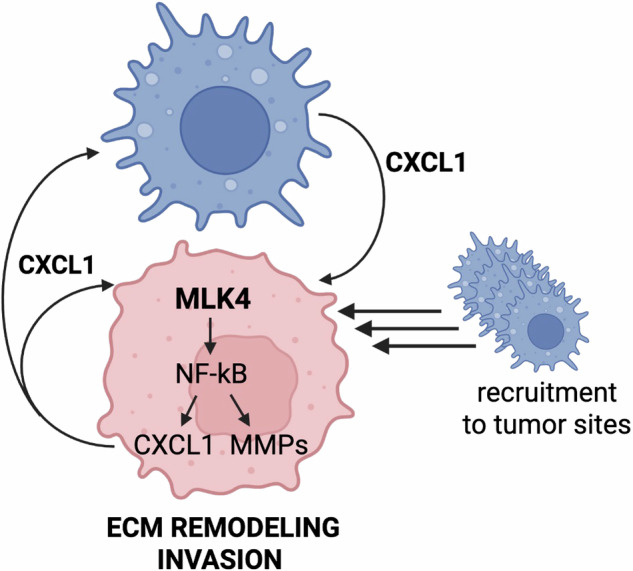
Schematic representation of TNBC–macrophage interactions regulated by the MLK4 signaling axis. High MLK4 expression in TNBC cells (pink) during cross-talk with macrophages (blue) promotes activation of the NF-κB signaling pathway, leading to increased secretion of MMPs and CXCL1, extracellular matrix (ECM) remodeling, and enhanced invasive activity of TNBC cells. This interaction also stimulates CXCL1 secretion by macrophages. Furthermore, elevated MLK4 expression in TNBC cells is associated with increased macrophage infi ltration into the tumor microenvironment (TME).

Interactions between breast cancer cells and macrophages induce a multitude of changes in cancer cells via different mechanisms. Here, we established the novel role of MLK4 in breast cancer cells as a critical mediator of the cross-talk between TAMs and TNBC. Our findings are consistent with earlier work, illustrating how MLK4 promotes aggressiveness of TNBC, but critically expand this paradigm to uncover the aspect of the cancer-promoting interactions with TAMs. Our study provides the first evidence that high MLK4 expression in TNBC cells is essential for TAMs to exert their pro-oncogenic influence on cancer cells. Using a co-culture system with both THP-1-derived M2 macrophages and primary hMDMs, we demonstrated that macrophages enhance the proliferation, migration, and invasive behavior of TNBC cells through an MLK4-dependent mechanism. Transcriptomic profiling and subsequent validation experiments revealed that MLK4 is vital for macrophage-induced activation of ECM remodeling, EMT, and MMP expression, which collectively contribute to increased TNBC invasiveness (Fig. 7). Mechanistically, our data support a model in which MLK4 drives TNBC aggressiveness via activation of signaling pathways such as NF-κB. We and others have previously shown that MLK4 activates the NF-κB signaling pathway, which regulates EMT, cytokine secretion and response to chemotherapy [21, 24, 25]. In line with this, we observed that NF-κB phosphorylation was increased by the presence of macrophages and significantly reduced in MLK4-deficient TNBC cells, further supporting a central role of this signaling axis in mediating macrophage-driven pro-tumorigenic effects.

Co-culture of TNBC cells with macrophages highlighted CXCL1 and IL-8 as the predominant cytokines secreted during their interaction. We also showed that CXCL1 is secreted by both macrophages and cancer cells, and is synergistically upregulated during co-culture. Importantly, silencing MLK4 in TNBC cells reduced CXCL1 expression and secretion, suggesting that CXCL1 is at least partially regulated by MLK4 signaling (Fig. 7). Notably, the top pathway enriched in our GO analysis is the glycosaminoglycan binding. This process takes part in modulating chemokine bioavailability and signaling duration. CXCL1 chemokine interacts with glycosaminoglycans on the surface of endothelial and epithelial cells and within the ECM [47]. This interaction stabilizes chemokines by protecting them from proteolytic degradation and vascular washout, thereby prolonging local signaling gradients [48]. In our study, CXCL1 markedly increased TNBC cell migration, whereas knockdown of MLK4 significantly attenuated this effect. These findings support a model in which chemokine binding to glycosaminoglycans could increase CXCL1 retention and signaling, thereby amplifying migratory cues in TNBC cells with high MLK4 expression, accelerating tumor progression. Although CXCL1 and IL-8 were identified as the primary factors enriched during TNBC-macrophages co-culture, the extracellular environment is far more complex and shaped by dynamic and multifactorial signaling molecules. Other factors may also contribute to this cross-talk, and further studies will be required to identify and characterize these molecular components.

Our data also show that high MLK4 expression correlates with increased macrophage infiltration in TNBC patient samples, suggesting that MLK4 may regulate macrophage recruitment to the tumor microenvironment. In this context, assessing MLK4 expression in cohorts of TNBC patients could provide prognostic value regarding immunotherapy response or macrophage-reprogramming therapeutics. Moreover, our findings raise the possibility that MLK4 signaling may extend beyond macrophage-TNBC interactions to influence other components of the TME, a possibility that requires further investigation.

Taken together, our results uncover novel functions of MLK4 signaling in the tumor microenvironment and provide a foundation for further exploration of MLK4-targeted therapies in TNBC. The development of inhibitors targeting MLK4 could provide a novel therapeutic strategy for TNBC patients, particularly those with macrophage-enriched tumors. From a therapeutic perspective, inhibiting MLK4 offers several potential advantages over currently pursued strategies aimed at downstream effectors such as NF-κB or MMPs. Although these pathways play central roles in cancer progression, clinical efforts to inhibit NF-κB or MMPs have largely failed due to broad pathway involvement in normal physiology, resulting in unacceptable off-target effects and toxicity [49, 50]. In contrast, MLK4 is markedly upregulated in TNBC, and this cancer-specific overexpression and its critical role in coordinating macrophages and TNBC cross-talk make MLK4 an attractive therapeutic target. Future studies will focus on the development of selective small-molecule inhibitors targeting MLK4 as a novel therapeutic strategy in TNBC.

## Supplementary information

Supplementary Information File

Uncropped blots

## Data Availability

All mRNA-seq data have been deposited at GEO DataSets GSE304746.
