# Supplementary material for: MLK4 orchestrates macrophage-induced triple-negative breast cancer invasion and ECM remodeling via enhanced paracrine signaling and NF-κB-MMP axis activation
Source: Cell Death Dis. 2026 Apr 1;17(1):440. doi: 10.1038/s41419-026-08689-y (PMC13168323; doi:10.1038/s41419-026-08689-y)
Supplement: Supplementary file 1 — Supplementary Information File [file 41419_2026_8689_MOESM1_ESM.docx]

**Supplementary Information file**

**MLK4 orchestrates macrophage-induced triple-negative breast cancer invasion and ECM remodeling via enhanced paracrine signaling and NF-κB-MMP axis activation**

Alicja Mazan-Bury, Dawid Mehlich, Kamila Karpińska, Michał Łaźniewski, Achilleas Moschos, Vi Nguyen-Phuong Truong, Paweł Jańczak, Anna A. Marusiak

**Content:**

1. **Supplementary Methods**
2. **Supplementary Figures and Figure Legends**
3. **Supplementary Tables**
4. **Supplementary References**

| **Main figures** | **Corresponding supplementary figures** |
| --- | --- |
| Fig. 1 | Supplementary Fig. 1  Supplementary Fig. 2  Supplementary Fig. 3  Supplementary Fig. 4 |
| Fig. 2 | Supplementary Fig. 5  Supplementary Fig. 6 |
| Fig. 3 | Supplementary Fig. 7 |
| Fig. 4 | Supplementary Fig. 8  Supplementary Fig. 9  Supplementary Fig. 10 |
| Fig. 5 | Supplementary Fig. 11 |
| Fig. 6 | Supplementary Fig. 12 |

**SUPPLEMENTARY MATERIALS AND METHODS**

**Generation of doxycycline-inducible cell lines**

Parental cell lines HCC1806 and SUM149PT were used to generate cells with doxycycline-inducible knock-down of MLK4, as described previously (1,2). Briefly, to generate lentiviral stock, HEK293T cells were transfected with TRIPZ shRNA_2 and shRNA_6 targeting MLK4 (Supplementary Table 1), purchased from Dharmacon. Cells were transduced with lentiviral stocks and subsequently selected with puromycin (Sigma). To induce knock-down of MLK4, cells were incubated with doxycycline (Sigma) at 1 µg/ml concentration for 48 h.

**siRNA transfection**

JetPRIME (Polyplus Transfections, #101000046) was used for siRNA transfections according to the manufacturer’s instructions. MLK4 silencing siRNA smart-pool (siRNA 1-4) and control siRNA (siGENOME Non-Targeting siRNA) were purchased from Dharmacon (Supplementary Table 1).

**Protein lysates preparation and immunoblotting**

For the whole cell lysates preparation, cells were lysed with Cell Lysis Buffer (Cell Signaling Tech., #9803S) supplemented with protease and phosphatase inhibitor tablets (Thermo Scientific™, #78444). Proteins were resolved by SDS-PAGE gel electrophoresis and transferred onto nitrocellulose membranes (Thermo Scientific™, #88018). Membranes were blocked in 5% fat-free milk in TBST buffer and incubated overnight at 4˚C with indicated primary antibodies, and then for 1 h with appropriate horseradish peroxidase–labeled secondary antibodies (Bio-Rad) (Supplementary Table 2). The blots were developed with Clarity (Bio-Rad, #1705061) and Clarity Max substrates (Bio-Rad, #1705062) and visualized using Amersham™ ImageQuant™ 800 (Cytiva). For densitometry, images were analyzed using ImageJ. As a loading control, membranes were incubated with a monoclonal anti-GAPDH antibody (Supplementary Table 2). Uncropped original western blots are in Supplementary Information File 2.

**Flow cytometry**

Cells were trypsinized and stained using the antibodies listed in Supplementary Table 2. Briefly, cells were washed with PBS and incubated with TruStain FcX (BioLegend, #422302) to block Fc receptors.  Fluorochrome-conjugated antibodies against CD14, CD80 and CD206 (Supplementary Table 2) were used for detection of surface markers. Samples were analyzed by the flow cytometry (LSR Fortessa) in the Laboratory of Cytometry, Nencki Institute of Experimental Biology, Polish Academy of Sciences. For flow cytometry data analysis BD FACSDiva software (BD Biosciences) was used.

**RT-qPCR**

Cells were lysed and RNA was isolated using GeneMATRIX Universal RNA Purification Kit (EURx, #E3598-02) according to the manufacturer's instructions. High-Capacity cDNA Reverse Transcription Kit (Applied Biosystems™, #4368814) was used for reverse transcription according to the manufacturer's instructions. iTaq™ Universal SYBR Green Supermix (Bio-Rad, #1725121) was used for RT-qPCR reaction. RT-qPCR reactions were performed on 96-well plates (Axygen, #PCR-96-FS-C), using Light Cycler 480 instrument (Roche). Samples were assayed in duplicates or triplicates, and the obtained Ct values were used to calculate relative gene expression using the 2−ΔCt method. For each gene, the expression was normalized to the expression of GAPDH, beta-actin or RPL37A housekeeping genes. The primers used in this study are listed in Supplementary Table 3.

**Gelatin Zymography**

TNBC cells were co-cultured with M2 macrophages or hMDMs for 48 h. Then Transwell inserts with macrophages were discarded, the media were changed for fresh and the TNBC cells were cultured for additional 24 h. Next, conditioned media were collected and centrifuged at 1,200 rpm, 4°C for 5 min to remove cell debris. Proteins secreted to the conditioned media were resolved by SDS-PAGE gel electrophoresis (1 mg/ml of gelatin in the gels). Following electrophoresis, gels were washed 2 times in renaturation buffer (5 mM CaCl_2_, 1 μM ZnCl_2_, 2.5% Triton X-100 in 50 mM Tris–HCl (pH 7.5)) for 1 h. Washed zymograms were incubated for 15 h at 37°C in incubation buffer (5 mM CaCl_2_, 1 μM ZnCl_2_, 1% Triton X-100 in 50 mM Tris–HCl (pH 7.5)). Then, gels were stained with Coomassie blue and destained with 40% methanol and 10% acetic acid. Areas of enzymatic activity appeared as clear bands over the dark background.

**Cytokine Array**

Proteome Profiler Human Cytokine Array Kit (Biotechne, #ARY005B) was used to determine secreted cytokine profiles. After 24 h, conditioned media were collected, from mono-culture of M2 macrophages, hMDMs, TNBC cells and co-culture of macrophages with TNBC cells. Then conditioned media were centrifuged at 1,200 rpm, 4°C for 5 min to remove cell debris, and used according to manufacturer instructions. The membranes were imaged using Amersham™ ImageQuant™ 800 (Cytiva).

**ELISA**

Conditioned media from mono-culture of M2 macrophages, hMDMs, TNBC cells and co-cultures of macrophages with TNBC cells with high and low level of MLK4 were collected after 24 h, centrifuged at 1,200 rpm, 4°C for 5 min to remove cell debris. To measure CXCL1 and IL-8 chemokines Human GRO alpha (CXCL1) Elisa Kit (Invitrogen, #BMS2122) and Human IL-8 Elisa Kit (Invitrogen, #KHC0081) were used respectively according to manufacturer instructions. Results were measured by absorbance detection (OD 450 nm) using Synergy Neo Plate Reader (BioTek) and concentrations were calculated using standard curve prepared based on standard samples from the kits.

**Wound healing assay**

THP-1-derived M2 macrophages were generated in 0.4 μm pore size Transwell inserts (Corning, # 3412). TNBC cells were pre-seeded into 6-well plates with or without doxycycline. After attachment to the plate, TNBC were co-cultured with macrophages. Control conditions included TNBC cells grown without macrophages. After 24 h of co-culture, the monolayer of TNBC cells was scratched using a 200 µl pipette tip. Three pictures of every wound in every condition were taken at time points 0 and 24 h after the scratch. Quantitative analysis was performed using ImageJ.

### Library preparation and next-generation sequencing

SUM149PT cells were transfected in triplicates with non-targeting control siRNA (siNT) or MLK4-targeting siRNA (siMLK4). 24 h after transfection, cells were either left untreated or co-cultured with THP-1-derived M2 macrophages for additional 48 h. Next, RNA was isolated using the RNAeasy Mini Kit (Qiagen, #74104), according to the manufacturer’s instructions. Libraries were prepared using KAPA mRNA HyperPrep Kit (Kapa Biosciences, #08098123702), KAPA RNA HyperPrep Kit (Kapa Biosciences, #08098107702) and KAPA Unique Dual-Indexed Adapter Kit (Kapa Biosciences, #08861919702). The quality of the obtained libraries was tested using Bioanalyzer-2100 (Agilent) and High Sensitivity DNA kit (Agilent, #5067-4626), according to the manufacturer’s instructions. Pair-end sequencing was performed with the NovaSeq 6000 S1 Reagent Kit (200 cycles, Illumina, #20028318) using NovaSeq 6000 instrument (Illumina).

**RNA-seq data processing**

For data analysis, raw sequences were trimmed according to quality using Trimmomatic using default parameters, except MINLEN, which was set to 20. Trimmed sequences were mapped to the human reference genome provided by ENSEMBL, (version grch38_snp_tran) using Hisat2 with default parameters. Optical duplicates were removed using MarkDuplicates tool from GATK with default parameters except OPTICAL_DUPLICATE_PIXEL_DISTANCE set to 12000. Mapped reads were associated with transcripts from GRCh38 database with default parameters except –stranded set to “reverse”. Differentially expressed genes were selected using DESeq2 package. Fold change was corrected using apeglm. Overrepresentation of Gene Ontology (The Gene Ontology Consortium 2019) terms and Kyoto Encyclopedia of Genes and Genomes (KEGG) categories among the selected genes was assessed with clusterprofiler package. Significance of association between the identified gene set and KEGG pathways was further tested with pathway regulation score. GSEA analysis was performed with phenoTest package. All mRNA-seq data have been deposited at GEO DataSets.

**Immunofluorescent staining and confocal microscopy**

SUM149_sh2 cells growing on glass coverslips were washed with PBS and fixed with 4% PFA in PBS for 20 min. Cells were then permeabilized with 0.1% Triton X-100 in PBS for 15 mins, washed and blocked with blocking buffer containing 0.1% BSA, 2.5% goat serum, and 2.5% donkey serum in PBS for 1 h. The primary p-NF-κB antibody was added in blocking buffer and incubated in a humid chamber at 4°C overnight. Cells were washed and incubated with secondary antibody (Goat anti-Rabbit IgG, Alexa Fluor 488, Invitrogen). Next, the cells were washed, and coverslips were mounted (Fluoromount-G™ Mounting Medium, Invitrogen). All images were captured with Zeiss LSM 910 Confocal Microscope. Image analysis was performed using ImageJ.

For evaluation of xenograft tumors, HCC1806 xenografts tissues were dissected as previously described (procedures approved by the Local Ethics Committee at the University of Warsaw, 1035/2020 and carried out in accordance with the requirements of EU Directive 2010/63/EU and Polish Dz. U. poz. 266/15.01.2015 legislation) (2). Xenografts tumors were mounted in O.C.T. (Sakura Tissue-Tek) and cut for thin sections using Leica CM1860 cryostat. Samples were permeabilized by incubation in 4% paraformaldehyde in phosphate-buffered saline followed by incubation with 0.1% Triton X-100 in PBS. Next, samples were blocked in blocking buffer (2,5% goat serum, 2,5% donkey serum, 0,1% bovine serum albumin, 0.1% Triton X-100 in PBS). CD68 antibody (Supplementary Table 2) was used to detect macrophages in xenografts tissues. Tissues were incubated overnight with primary CD68 antibody and then for 1 h with secondary antibody (Supplementary Table 2). Then, Tissues were mounted in Fluoromount-G Mounting Medium with DAPI (Supplementary Table 2), a blue nuclear stain. Stained xenografts tissues were visualized using Nikon Eclipse 80i Fluorescence Microscope.

**CIBERSORTx analysis**

RNAseq expression files for the Breast Invasive Carcinoma cohort (TCGABRCA) were downloaded from the GDC Data Portal in January 2023 (3). These counts were obtained by aligning reads to the human genome (GRCh38) with STAR and quantifying genes according to GENCODE v36. Sample metadata were used to classify tumors as (i) triple-negative breast cancer (TNBC) or (ii) non-TNBC. TNBC was defined as ER-negative, PR-negative, and HER2-negative infiltrating ductal carcinoma, following the filtering recipe of Craven et al. (4). This yielded 119 TNBC and 808 nonTNBC samples, consistent with the Craven cohort. A TPM matrix was prepared and submitted to CIBERSORTx (5). We used the LM22 signature matrix (547 genes, 22 immune populations) to estimate the abundances of immune cell types in TCGA samples. Additional parameters included 1,000 permutations, and disabled quantile normalization, as recommended for bulk RNA-seq data. Samples with a CIBERSORTx empirical p-value > 0.05 (n = 13) were excluded from further analysis. Fractional scores for each cell type were then normalized so that, for each sample, the sum of all cell-type fractions equaled 1. The CIBERSORTx monocyte and macrophage subsets (M0, M1, M2) were combined into a single group. All downstream analyses were performed in R 4.3.3 using a custom script. All tests were two-tailed with α = 0.05.

**SUPPLEMENTARY FIGURES AND FIGURE LEGENDS**

**Supplementary Figure 1. THP-1-derived M0, M1, M2 and TAM-like macrophage generation and validation. A-B.** THP-1 were treated with 150 nM PMA for 24 h, followed by a 24 h rest period with fresh culture medium. For M1, cells were incubated with 100 ng/mL LPS, and 20 ng/mL IFN-gamma for 18 h. For M2, cells were incubated with 20 ng/mL IL-4, and 20 ng/mL IL-13 for 48 h. For TAM-like macrophages, cells were incubated for 48 h with 30% conditioned media (CM) collected from the breast cancer cells. Following macrophage generation, cells were stained with M1 marker CD38-PE and M2 marker CD206-BV421, and analyzed by flow cytometry (A and B). Representative dot plots from flow cytometry analysis of M0, M1, M2 and TAM-like macrophages stained with CD38-PE and CD206-BV421 (A). Data represent mean results from three or more independent experiments (error bars ±SD) (B). Significance was calculated using one-way ANOVA followed by the Tukey multiple comparisons test. **C.** Following macrophage generation as described above, RNA was isolated, and relative gene expression of M1 markers (CXCL10 and TNF-alpha) and M2 markers (CD206 and CD163) was analyzed by RT-qPCR. Data represent mean results from three independent experiments (error bars ±SD). Significance was calculated using one-way ANOVA followed by the Tukey multiple comparisons test.


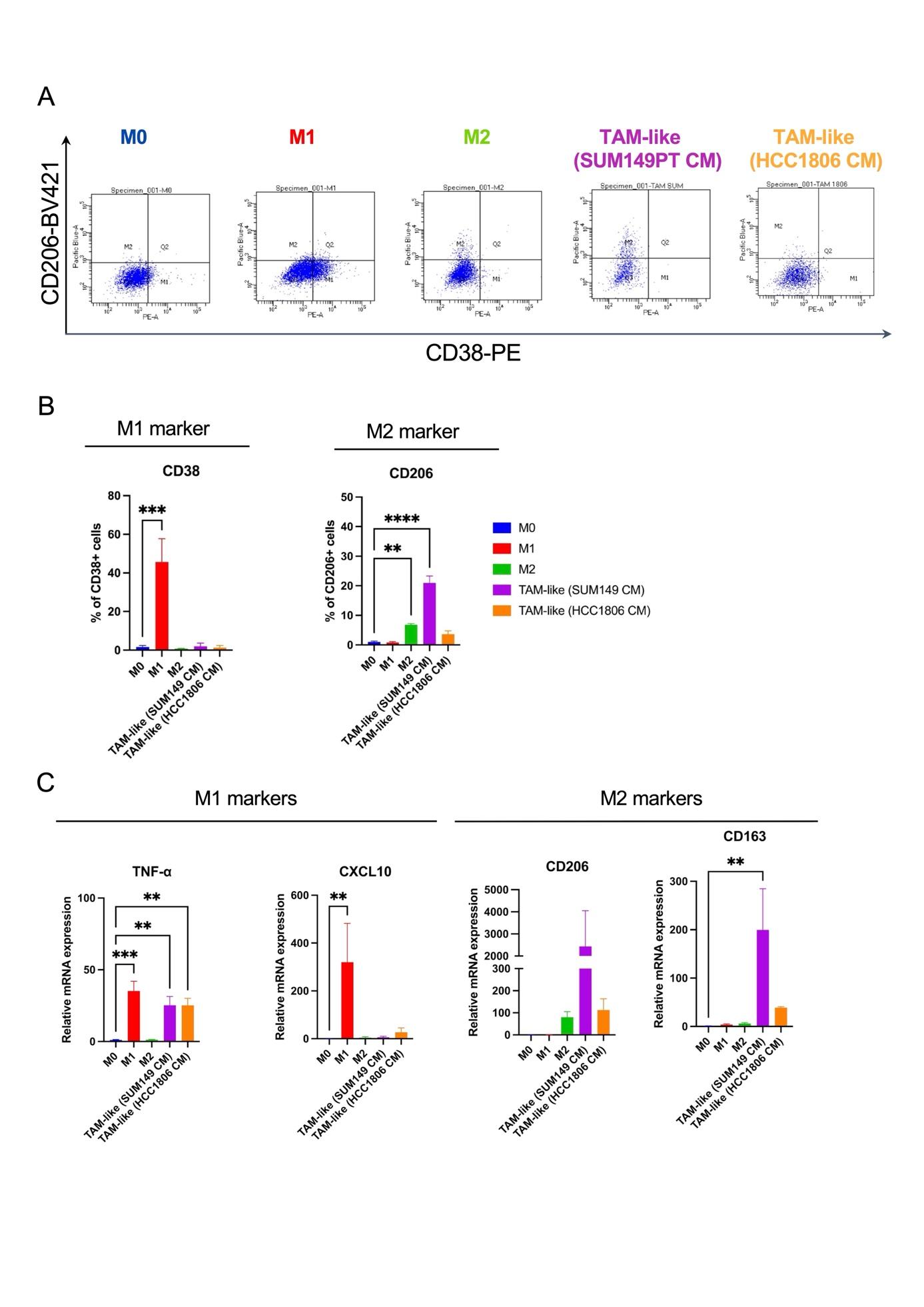


**Supplementary Figure 2. A.** Cell lines with doxycycline-inducible MLK4 knock-down were generated from SUM149PT and HCC1806 parental cells using lentiviral vectors. Silencing of MLK4 was confirmed by immunoblotting. **B.** THP-1-derived M2 (B) and TAM-like (C) macrophages were generated in 0.4 μm Transwell inserts. Briefly, THP-1 were treated with 150 nM PMA for 24 h, followed by a 24 h rest period with fresh culture media. For M2, cells were incubated with 20 ng/mL IL-4, and 20 ng/mL IL-13 for 48 h (B). For TAM-like macrophages, cells were incubated for 48 h with 30% conditioned media (CM) collected from the breast cancer cells (C). SUM149_sh6 and HCC1806_sh2 were pre-seeded into 6-well plates and treated with doxycycline to induce knock-down of MLK4. After 24 h, SUM149_sh6 and HCC1806_sh2 were co-cultured with M2 (B) or TAM-like macrophages (C). Control conditions included TNBC cells grown without macrophages (No CC). After 4 days of co-culture, TNBC cells were stained with crystal violet and pictures were taken. The proliferation rate was quantified by measuring the absorbance after solubilization of the dye (OD540 nm). Data represent mean results from at least three independent experiments (error bars ± SEM). Significance was calculated using two-way ANOVA followed by the Tukey multiple comparisons test.


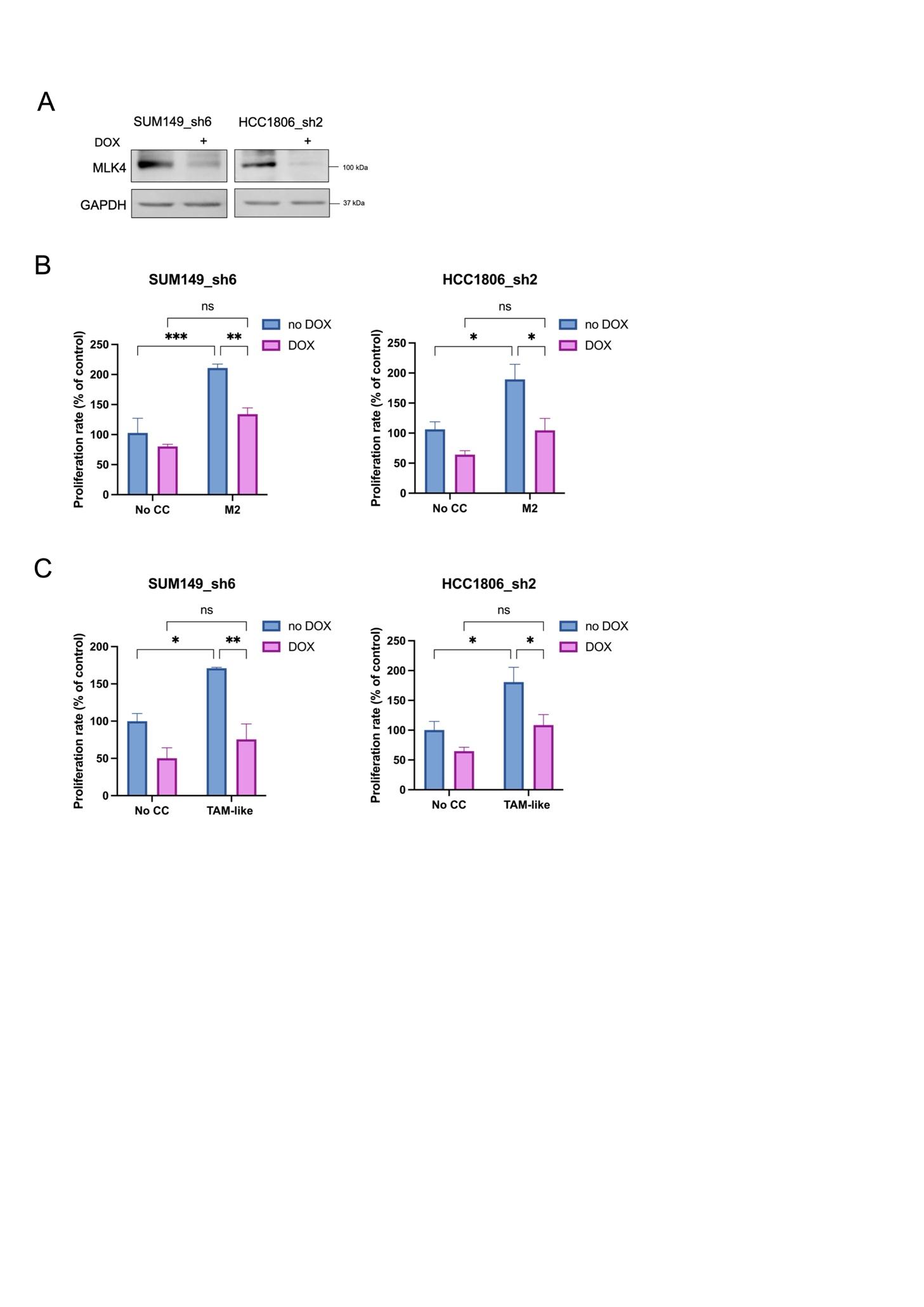


**Supplementary Figure 3.** THP-1derived M2 and TAM-like macrophages were generated in 0.4 μm Transwell inserts. Briefly, THP-1 were treated with 150 nM PMA for 24 h, followed by a 24 h rest period with fresh culture media. For M2, cells were incubated with 20 ng/mL IL4, and 20 ng/mL IL13 for 48 h. For TAM-like macrophages, cells were incubated for 48 h with 30% conditioned media (CM) collected from the breast cancer cells. Parental SUM149PT and HCC1806 were pre-seeded into 6-welll plates and treated with doxycycline. After 24 h, SUM149PT and HCC1806PT were co-cultured with M2 or TAM-like macrophages. Control conditions included TNBC cells grown without macrophages (No CC). After 4 days of co-culture, TNBC cells were stained with crystal violet and pictures were taken. The colony number was quantified by measuring the absorbance after solubilization of the dye (OD540 nm). Data represent mean results from at least three independent experiments (error bars ±SEM). Significance was calculated using two-way ANOVA followed by the Tukey multiple comparisons test.


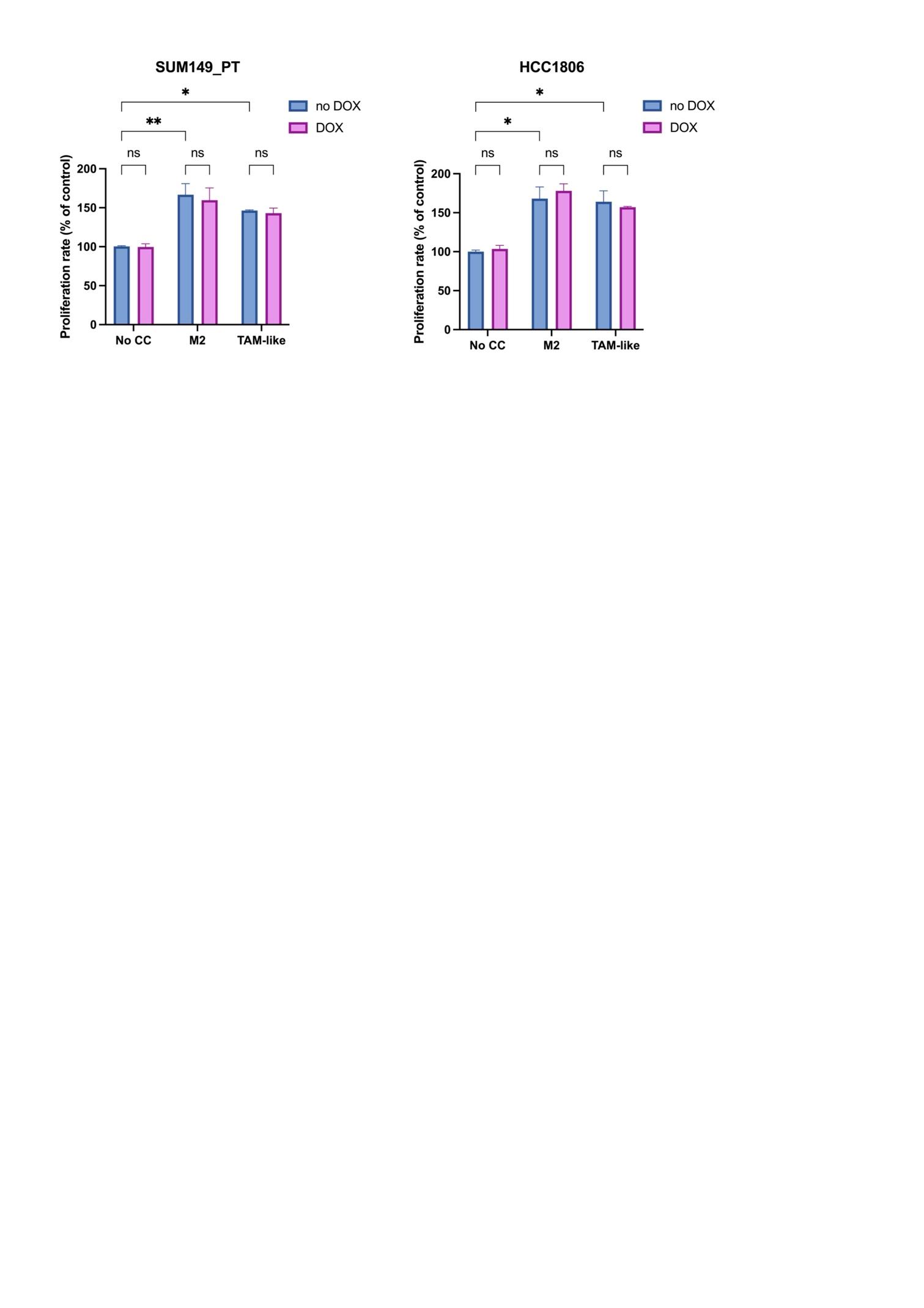


**Supplementary Figure 4.** THP-1derived M2 macrophages were generated in 0.4 μm Transwell inserts. Briefly, THP-1 were treated with 150 nM PMA for 24 h, followed by a 24 h rest period with fresh culture media. Then, cells were incubated with 20 ng/mL IL-4, and 20 ng/mL IL-13 for 48 h. SUM149PT_sh2 and HCC1806_sh6 were pre-seeded into 6-well plates and treated with doxycycline to induce knock-down of MLK4 for 48 h. SUM149PT_sh2 and HCC1806_sh6 cells were co-cultured with M2 for 24 h and then subjected to wound healing assay. Control conditions included TNBC cells grown without macrophages (No CC). Representative pictures are shown. Quantification was performed using ImageJ. Data represent mean results from at least three independent experiments (error bars ±SEM). Significance was calculated using two-way ANOVA followed by the Tukey multiple comparisons test.


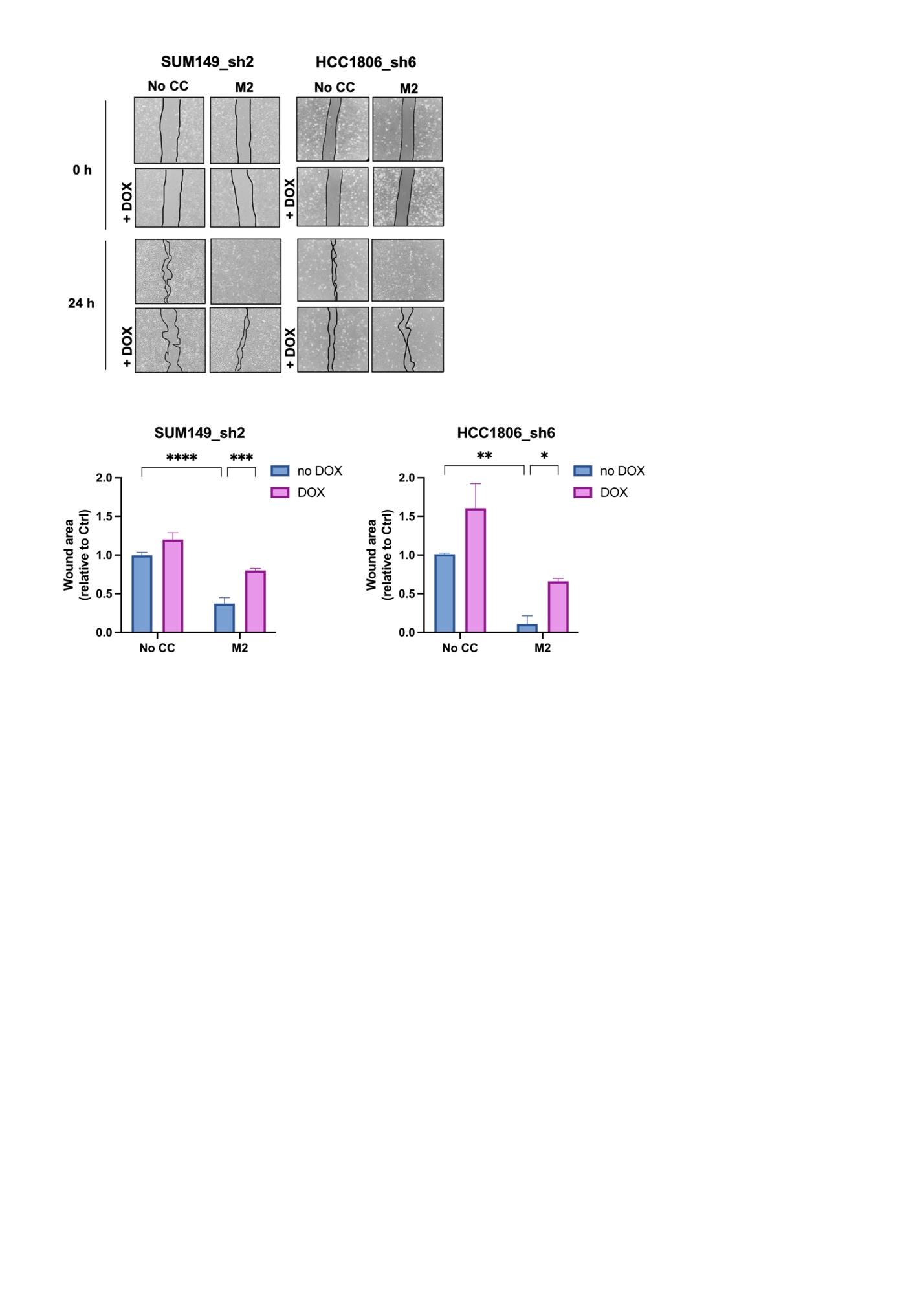


**Supplementary Figure 5.** Human monocyte-derived macrophages (hMDMs) generation and validation by flow cytometry. To generate mature hMDMs, peripheral blood mononuclear cells (PBMCs) were isolated from human healthy donors’ buffy coats using gradient medium Lymphoprep™ (StemCell), according to the manufacturer's instructions. Next, EasySep™ Human Monocyte Isolation Kit was used to isolate human CD14+ CD16- monocytes from PBMCs according to the manufacturer's protocol. Isolated monocytes were cultured in RPMI-1640 medium supplemented with 10% FBS, 1% penicillin/streptomycin, 2 mM L-glutamine and 20 ng/ml huGM-CSF. Every two days culture medium was refreshed. After 7 days of treatment with huGM-CSF cells were ready for validation. Mature hMDMs were dissociated using Enzyme-free Cell Dissociation Solution (ScienCell). Next, cells were washed with PBS and incubated with TruStain FcX to block Fc receptors. Fluorochrome-conjugated antibodies against CD14-AF488 (A), CD80-PE and CD206-BV421 (B and C) were used for detection of surface markers. Samples were analyzed using flow cytometer (BD LSR Fortessa Analyser). For data analysis BD FACSDiva software was used.


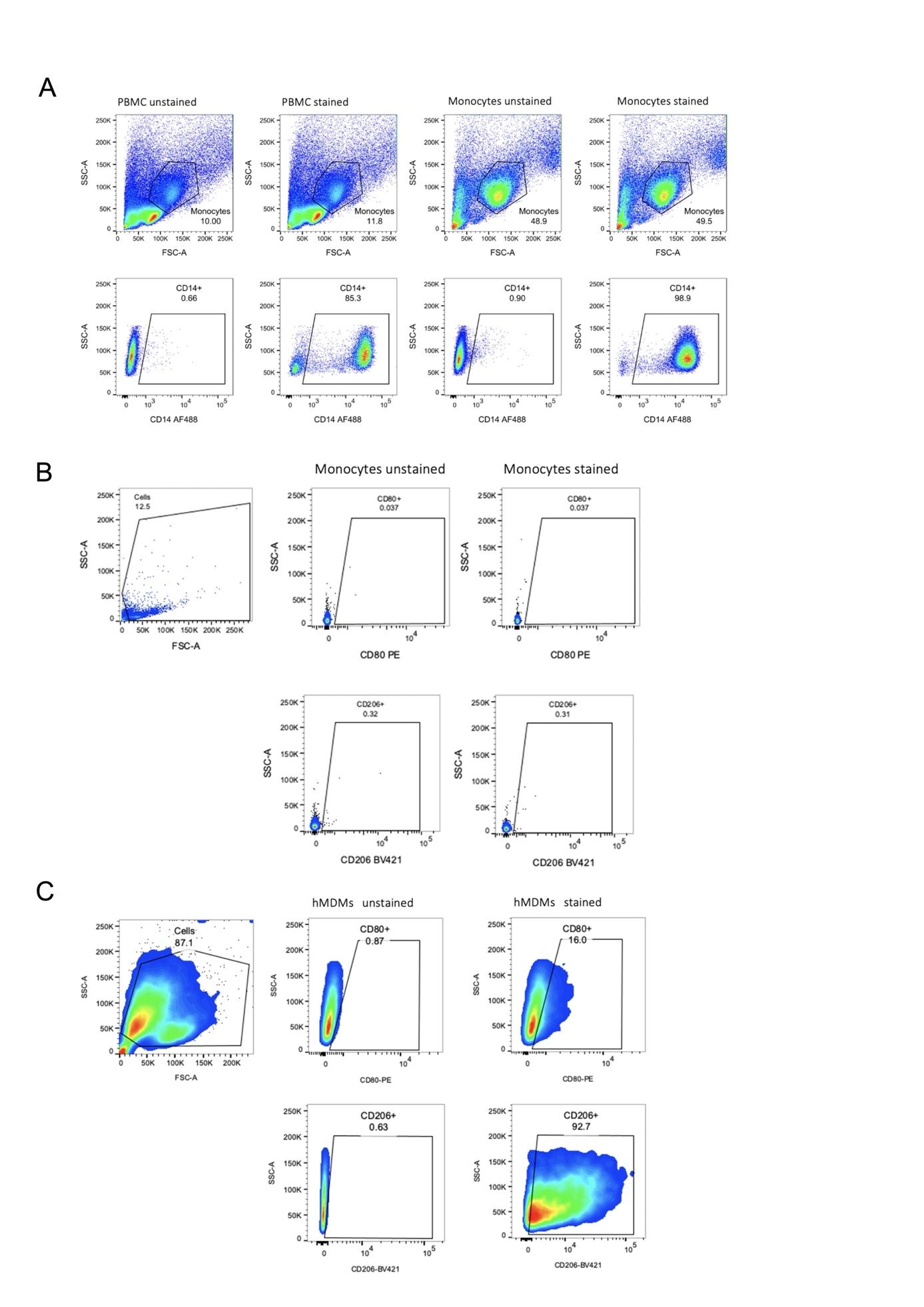


**Supplementary Figure 6.** Human monocyte-derived macrophages (hMDMs) generation and validation by qRT-PCR. hMDMS were generated as previously (Supplementary Fig. 5). Mature hMDMs were dissociated using Enzyme-free Cell Dissociation Solution (ScienCell). Next, cells were washed with PBS. Cells were isolated and RNA was isolated and relative gene expression of M1 marker (TNF-alpha) and M2 marker (CD206) was analyzed by RT-qPCR. For each gene, the expression was normalized to the expression of GAPDH, beta-actin or RPL37A housekeeping genes. Significance was calculated using one-way ANOVA followed by the Tukey multiple comparisons test.


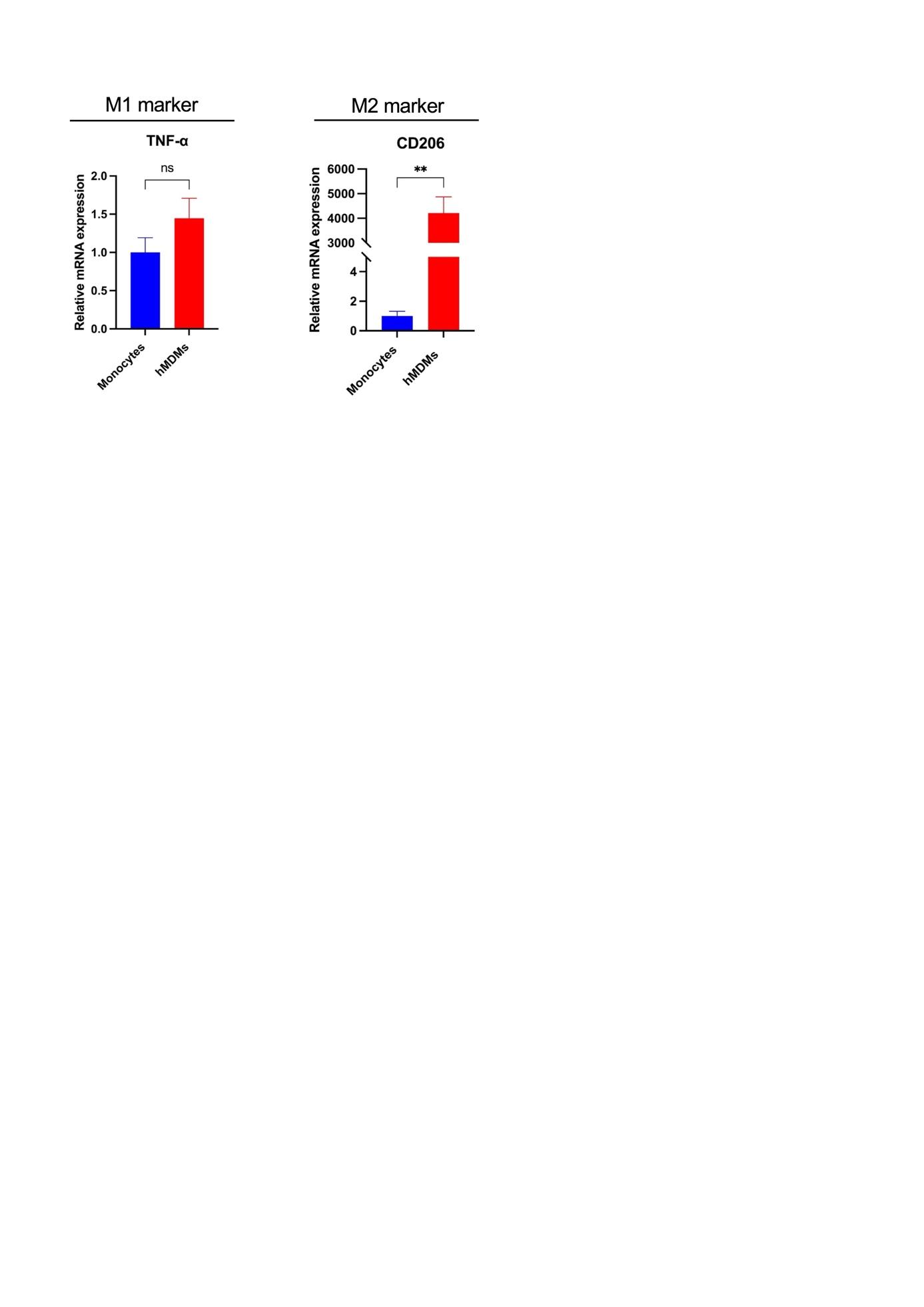


**Supplementary Figure 7.** **A.** Mean normalized counts for MLK4 transcripts from mRNA-seq. **B.** Principal component analysis plot obtained after mRNA-seq of SUM149PT transfected with MLK4-targeting siRNA or control siRNA (siNT), co-cultured or not with M2 macrophages.


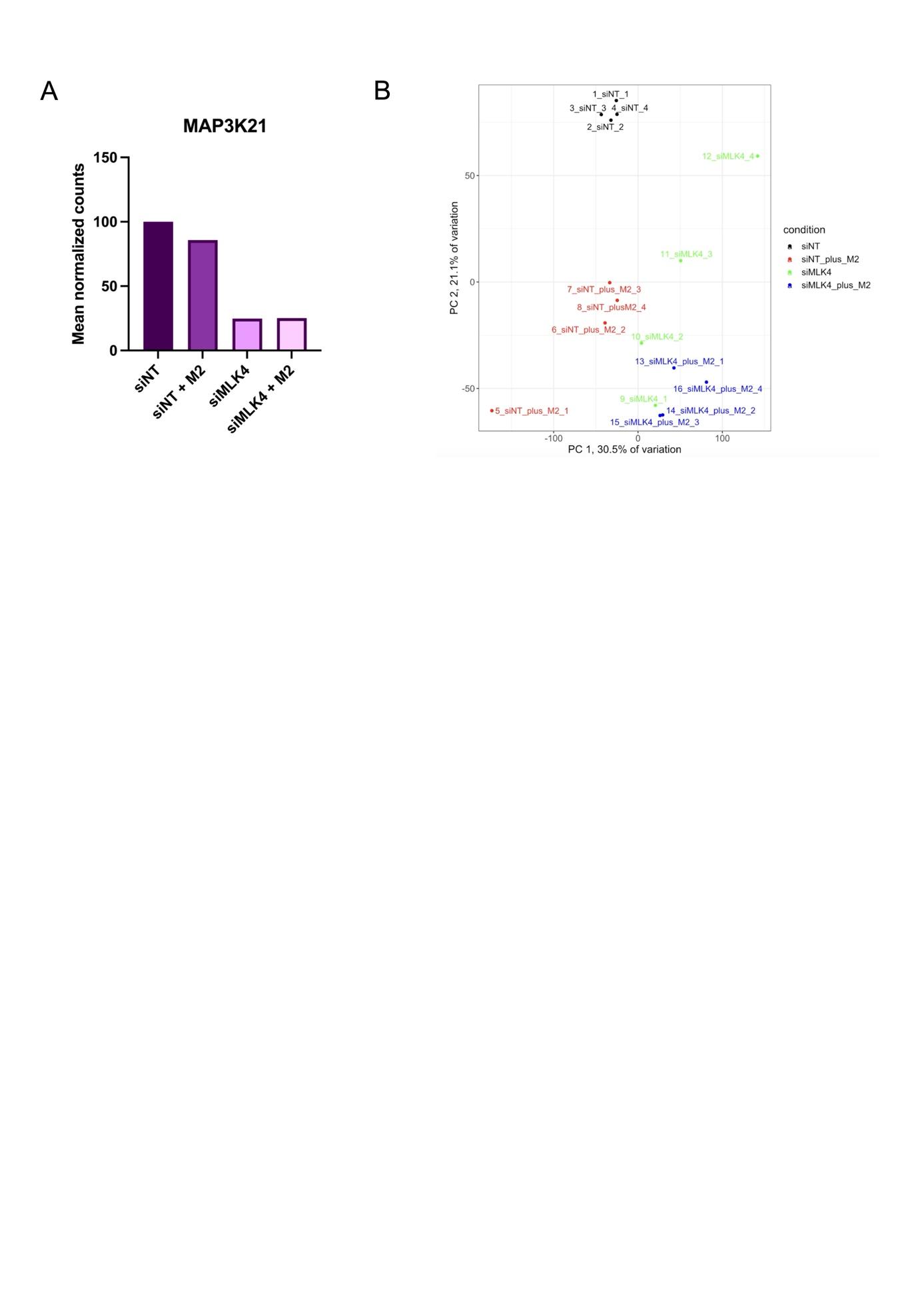


**Supplementary Figure 8.** SUM149PT cells were transfected with non-targeting siRNA (siNT) or MLK4-targeting siRNA (siMLK4). Following the transfection, cells were co-cultured with THP-1-derived M2 macrophages for 24 h. The RNA was isolated and relative gene expression of MMP9 was analyzed by RT-qPCR (error bars ±SEM). Significance was calculated using two-way ANOVA followed by the Tukey multiple comparisons test.


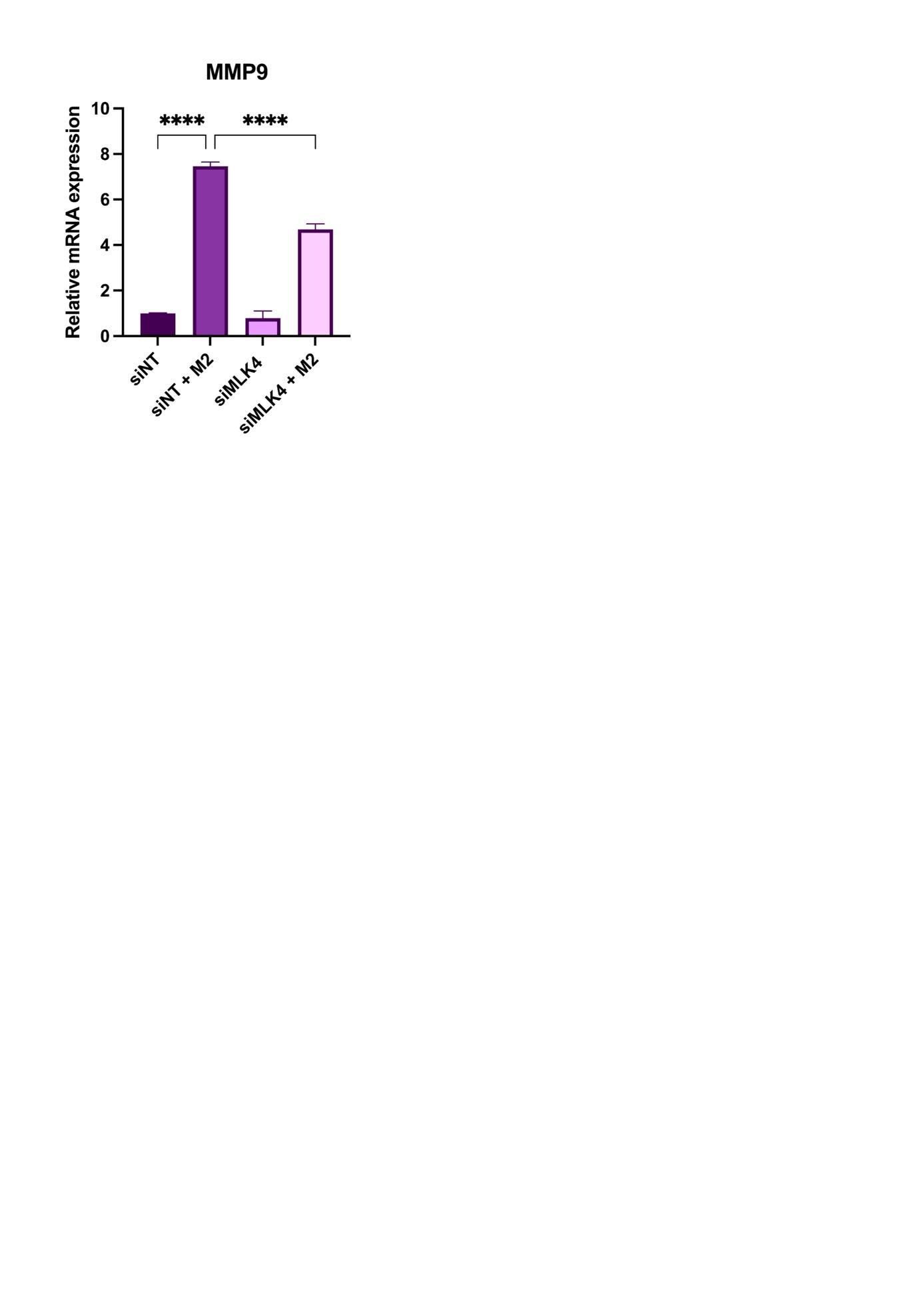


**Supplementary Figure 9.** SUM149PT cells were transfected with non-targeting siRNA (siNT) or MLK4-targeting siRNA (siMLK4). Following the transfection, cells were co-cultured with THP-1-derived M2 macrophages or hMDMs for 48 h. After the co-culture period, we separated TNBC cells from macrophages, replaced the media, and collected the conditioned supernatants (after 24 h) for gelatin zymography. TNBC cells were lysed and whole cell lysates were analyzed by immunoblotting.


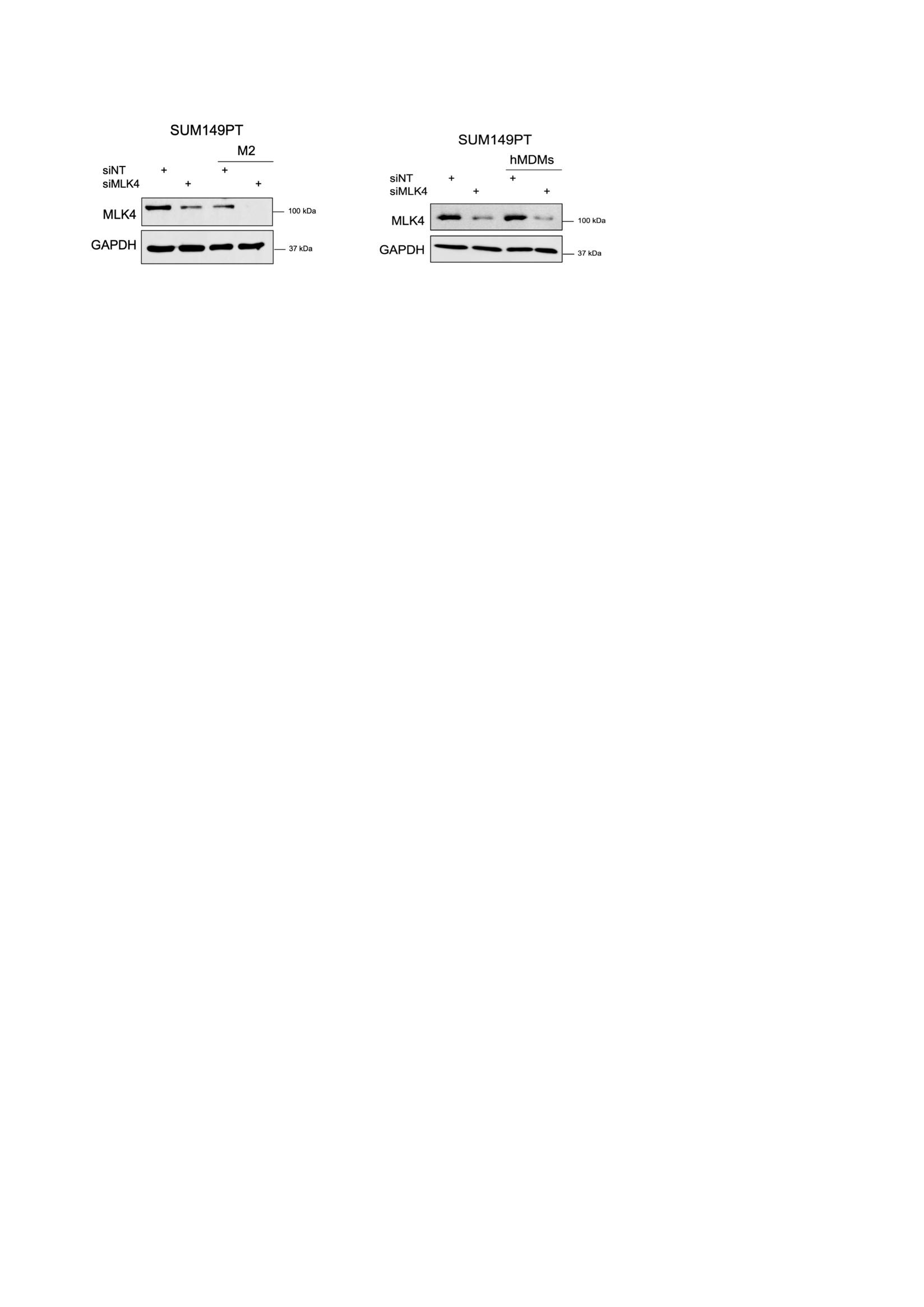


**Supplementary Figure 10.** **A.** Representative confocal images of SUM149_sh2 cells monocultured or co-cultured with macrophages and stained for p-NF-κB. SUM149_sh2 were pre-seeded into 6-well plates and treated with doxycycline to induce knock-down of MLK4. hMDMS were generated in inserts as described previously and added to the wells with TNBC cells for co-culture for 24 h. Following co-culture, SUM149_sh2 cells were stained with p-NF-κB (AF488, green). SUM149_sh2 cells with dox-induced MLK4 knock-down express red-fluorescence protein (RFP). Nuclei were stained with DAPI (blue). Scale bar 10 μm. **B.** Quantification of AF488 intensity measured for 19 or more individual cells per condition (error bars ±SEM). Significance was calculated using one-way ANOVA followed by the Tukey multiple comparisons test.


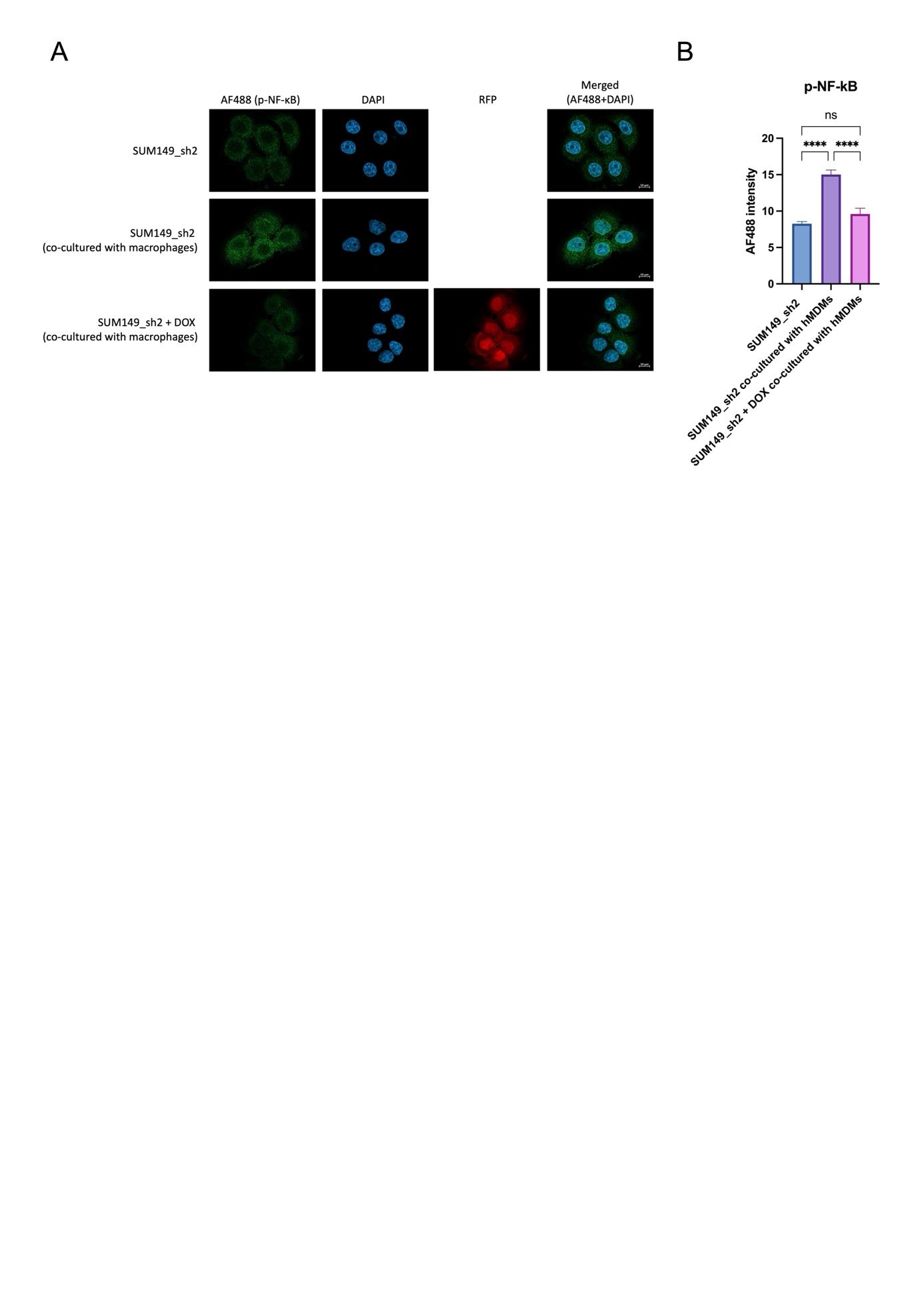


**Supplementary Figure 11.** **A.** Human Cytokine Array (Biotechne, #ARY005B) layout. **B.** THP-1-derived M2 macrophages were generated in 0.4 μm Transwell inserts. Briefly, THP-1 were treated with 150 nM PMA for 24 h, followed by a 24 h rest period with fresh culture media. Then, cells were incubated with 20 ng/mL IL-4, and 20 ng/mL IL-13 for 48 h. hMDMs were generated in 0.4 μm Transwell inserts. SUM149_sh2 were pre-seeded into 6-well plates and treated with doxycycline to induce knock-down of MLK4. After 24 h, inserts with M2 and hMDMs were added to the wells with TNBC cells. Control conditions included SUM149_sh2, M2 and hMDMs mono-cultures. Supernatants from mono-cultures and co-cultures were collected after 24 h and subjected to ELISA assay (error bars ±SEM). Significance was calculated using one-way ANOVA followed by the Tukey multiple comparisons test.


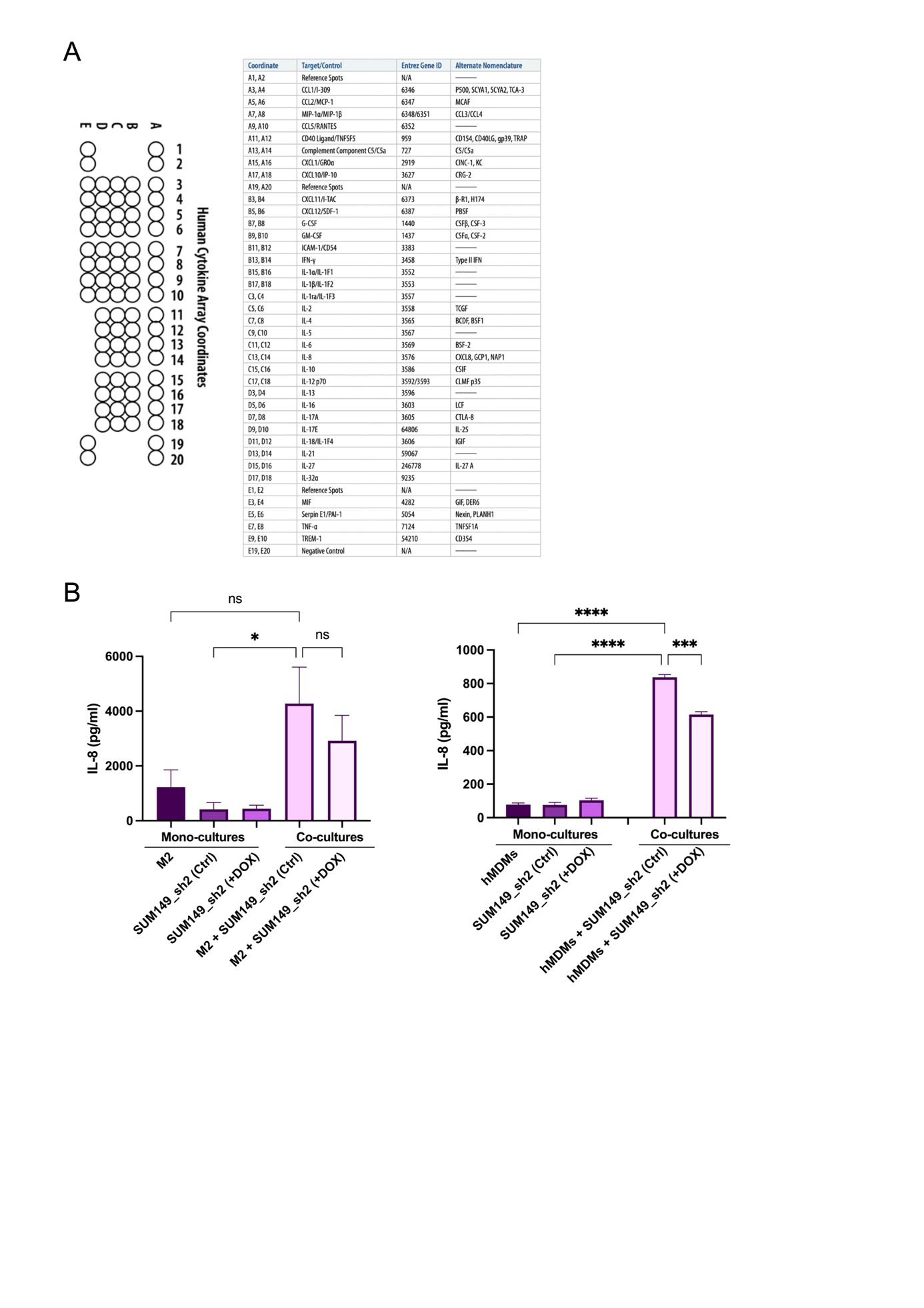


**Supplementary Figure 12.** SUM149_sh2 and HCC1806_sh6 cells were seeded into 24-well plates and pre-treated with doxycycline to induce MLK4 knock-down for 48 h. THP-1 were seeded into 8 μm pore size Transwell inserts and treated with 150 nM PMA. After 24 h inserts were placed into the wells with or without SUM149_sh2 or HCC1806_sh6. Control conditions included THP-1-derived macrophages seeded into 8 μm pore size Transwell inserts and placed into the wells without TNBC cells (No CC). After 24 h, THP-1 macrophages that migrated were stained with crystal violet and the pictures were taken. Results were quantified by ImageJ. Data represent mean results from three independent experiments (error bars ±SEM). Significance was calculated using two-way ANOVA followed by the Tukey multiple comparisons test.


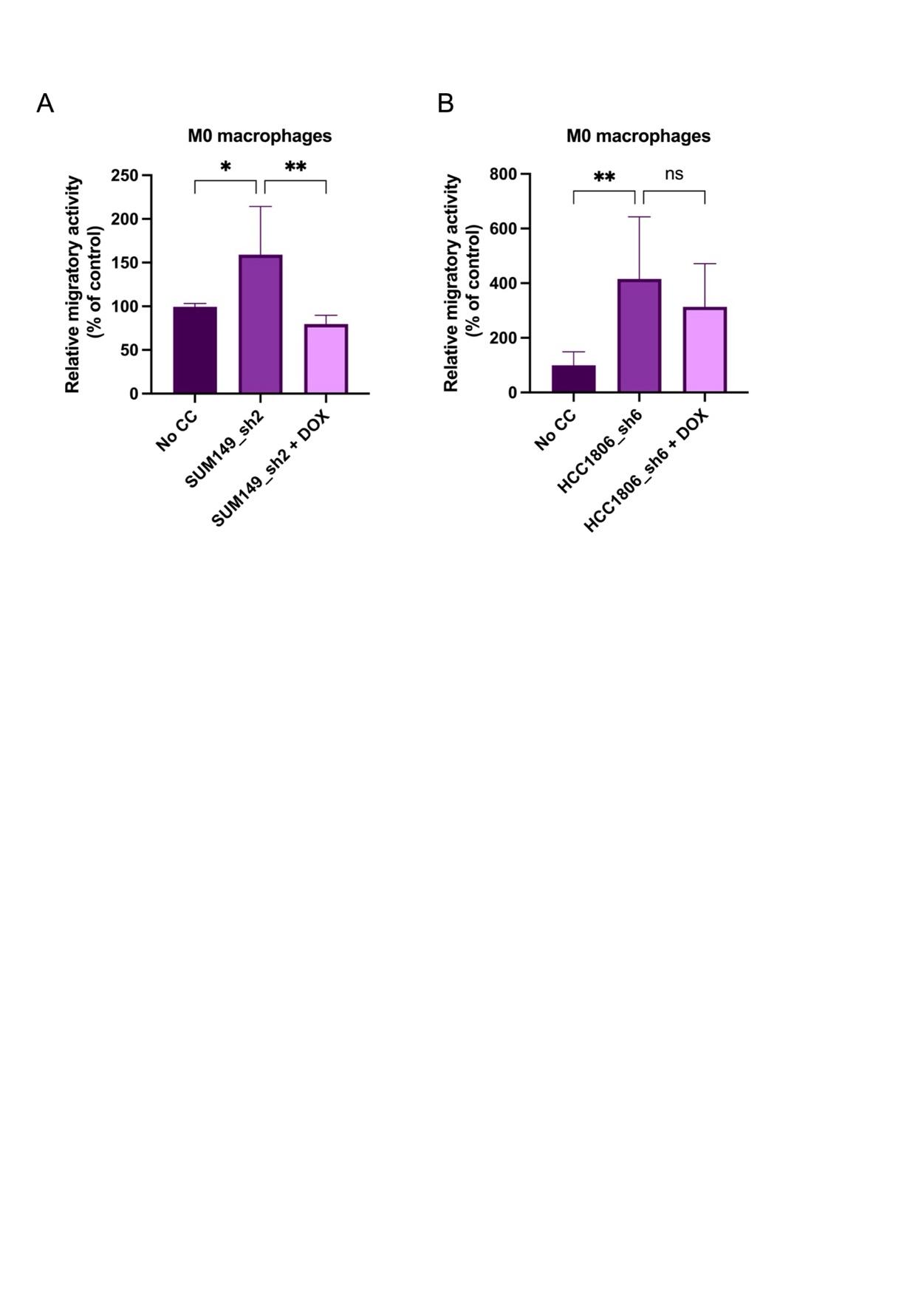


**SUPPLEMENTARY TABLES**

**Supplementary Table 1. List of siRNA and shRNA sequences.**

| **siRNA** | **Sequence** |
| --- | --- |
| siMLK4 #1 | GGAAAGAUGCUCAGAGAGAUU |
| siMLK4 #2 | AGGAGAAGCCCAAGGUAAAUU |
| siMLK4 #3 | AGAAGAAACGAGAGGGAAUUU |
| siMLK4 #4 | AGAACAGAUUGCAAAGAAAUU |
| **shRNA** | **Sequence** |
| sh_2 | ATCAGAATGTTAAGTTCCC |
| sh_6 | TCTTGATACACTACAATCA |

**Supplementary Table 2. List of antibodies used in this study.**

| **Antibody name** | **Company** | **Catalog number** |
| --- | --- | --- |
| CD68 (IF – primary antibody) | Abcam | #ab303565 |
| DAPI (IF) | Invitrogen™ | #00-4959-52 |
| Alexa Fluor™ 488 goat anti-rabbit IgG (IF-secondary antibody) | Invitrogen™ | #A32731 |
| Alexa Fluor™ 594 donkey anti-rabbit IgG  (IF – secondary antibody) | Invitrogen™ | #A-21207 |
| CD206-BV421  (Flow cytometry) | BD Biosciences | #564062 |
| CD38-PE  (Flow cytometry) | BD Biosciences | #560981 |
| CD14-AF488  (Flow cytometry) | BD Biosciences | #557700 |
| CD80-PE  (Flow cytometry) | BioLegend | #375409 |
| GAPDH  (Immunoblotting) | Cell Signalling Technology | #2118 |
| MLK4  (Immunoblotting) | Bethyl Laboratories | #A302-610A |
| phospho-NF-κB  (Immunoblotting and IF – primary antibody) | Cell Signalling Technology | #3033 |
| NF-κB  (Immunoblotting) | Cell Signalling Technology | #8242 |
| Vimentin  (Immunoblotting) | Cell Signalling Technology | #5741 |
| Snail  (Immunoblotting) | Cell Signalling Technology | #3879 |
| MMP28  (Immunoblotting) | Protein Tech | #18237-1-AP |
| ADAM12  (Immunoblotting) | Protein Tech | #14139-1-AP |
| MMP9  (Immunoblotting) | Cell Signalling Technology | #13667 |

**Supplementary Table 3. List of primers used in this study.**

| **Gene** | **Forward Primer (5’-3’)** | **Reverse Primer (3’-5’)** |
| --- | --- | --- |
| ADAM12 | GCAGTTTCACGGAAACCCAC | ACACGTGCTGAGACTGACTG |
| CCN4 (WISP1) | CTTGGAATCCTACCCTGACTTCT | CTTCACAGGCATTGGGTTAGTC |
| CD163 | TTTGTCAACTTGAGTCCCTTCAC | TCCCGCTACACTTGTTTTCAC |
| CD206 | CCATGGACAATGCGCGAGCG | CACCTGTGGCCCAAGACACGT |
| CRABP2 | CTGGGGGTGAATGTGATGCT | CCTCAAACTCCTCCCCAACC |
| CXCL1 | AGCTTGCCTCAATCCTGCATCC | TCCTTCAGGAACAGCCACCAGT |
| CXCL10 | GAAAGCAGTTAGCAAGGAAAGGTC | ATGTAGGGAAGTGATGGGAGAGG |
| GAPDH | CCATGGAGAAGGCTGGGG | GTCCACCACCCTGTTGCTGTA |
| IL13RA2 | TAAACCTTTGCCGCCAGTCT | AGGTCCCAAAGGTATGCTCC |
| MLK4 | CATGAGGAGGCCTTCGTG | CGCCAACCCAAAATCTGTAA |
| MMP7 | TCCAACCTATGGAAATGGAGA | GGAGTGGAGGAACAGTGCTT |
| MMP9 | TTCCAAACCTTTGAGGGCGA | CAAAGGCGTCGTCAATCACC |
| NDRG1 | TGACATGACTAGTGGCTCTTCT | CAGTCAGCTTACCAAGCATACT |
| RPL37A | AGCTGTGGGGATCTGGCACT | CGTGACAGCGGAAGTGGTATTGTA |
| TNF-α | CTCTTCTGCCTGCTGCACTTTG | ATGGGCTACAGGCTTGTCACTC |
| β-actin | CATCCTCACCCTGAAGTACC | AGCCTGGATAGCAACGTACAT |

**SUPPLEMENTARY REFERENCES**

1. Marusiak AA, Prelowska MK, Mehlich D, Lazniewski M, Kaminska K, Gorczynski A, et al. Upregulation of MLK4 promotes migratory and invasive potential of breast cancer cells. Oncogene. 2019 Apr;38(15):2860–75.

2. Mehlich D, Łomiak M, Sobiborowicz A, Mazan A, Dymerska D, Szewczyk ŁM, et al. MLK4 regulates DNA damage response and promotes triple-negative breast cancer chemoresistance. Cell Death Dis. 2021 Nov 27;12(12):1111.

3. Heath AP, Ferretti V, Agrawal S, An M, Angelakos JC, Arya R, et al. The NCI Genomic Data Commons. Nat Genet. 2021 Mar;53(3):257–62.

4. Craven KE, Gökmen-Polar Y, Badve SS. CIBERSORT analysis of TCGA and METABRIC identifies subgroups with better outcomes in triple negative breast cancer. Sci Rep. 2021 Feb 25;11(1):4691.

5. Newman AM, Liu CL, Green MR, Gentles AJ, Feng W, Xu Y, et al. Robust enumeration of cell subsets from tissue expression profiles. Nat Methods. 2015 May;12(5):453–7.
