# Supplementary material for: MLK4 orchestrates macrophage-induced triple-negative breast cancer invasion and ECM remodeling via enhanced paracrine signaling and NF-κB-MMP axis activation
Source: Cell Death Dis. 2026 Apr 1;17(1):440. doi: 10.1038/s41419-026-08689-y (PMC13168323; doi:10.1038/s41419-026-08689-y)
Supplement: Supplementary file 2 — Uncropped blots [file 41419_2026_8689_MOESM2_ESM.pptx]

## Slide 1
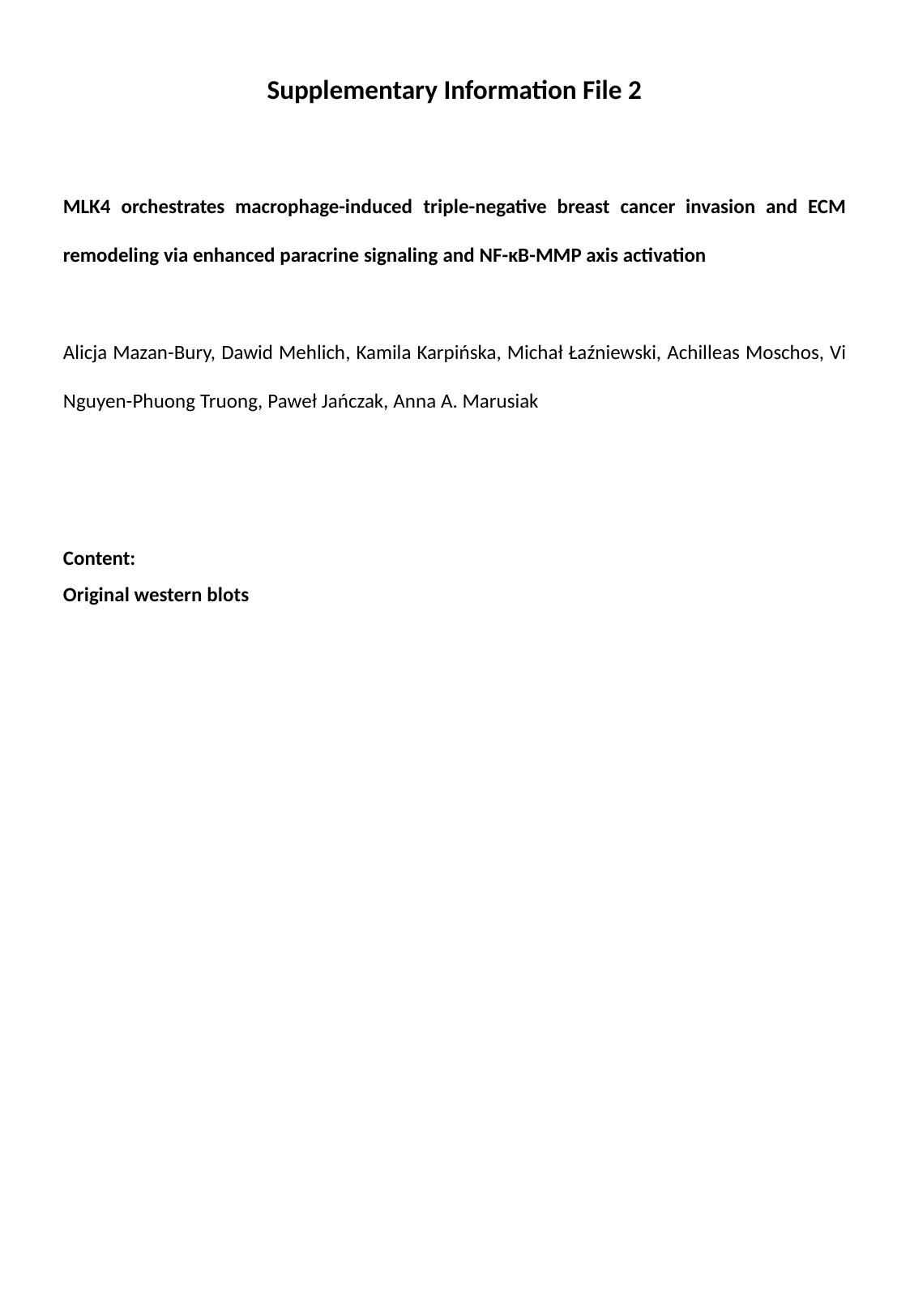

Supplementary Information File 2
MLK4 orchestrates macrophage-induced triple-negative breast cancer invasion and ECM remodeling via enhanced paracrine signaling and NF-κB-MMP axis activation
Alicja Mazan-Bury, Dawid Mehlich, Kamila Karpińska, Michał Łaźniewski, Achilleas Moschos, Vi Nguyen-Phuong Truong, Paweł Jańczak, Anna A. Marusiak
Content:
Original western blots

## Slide 2
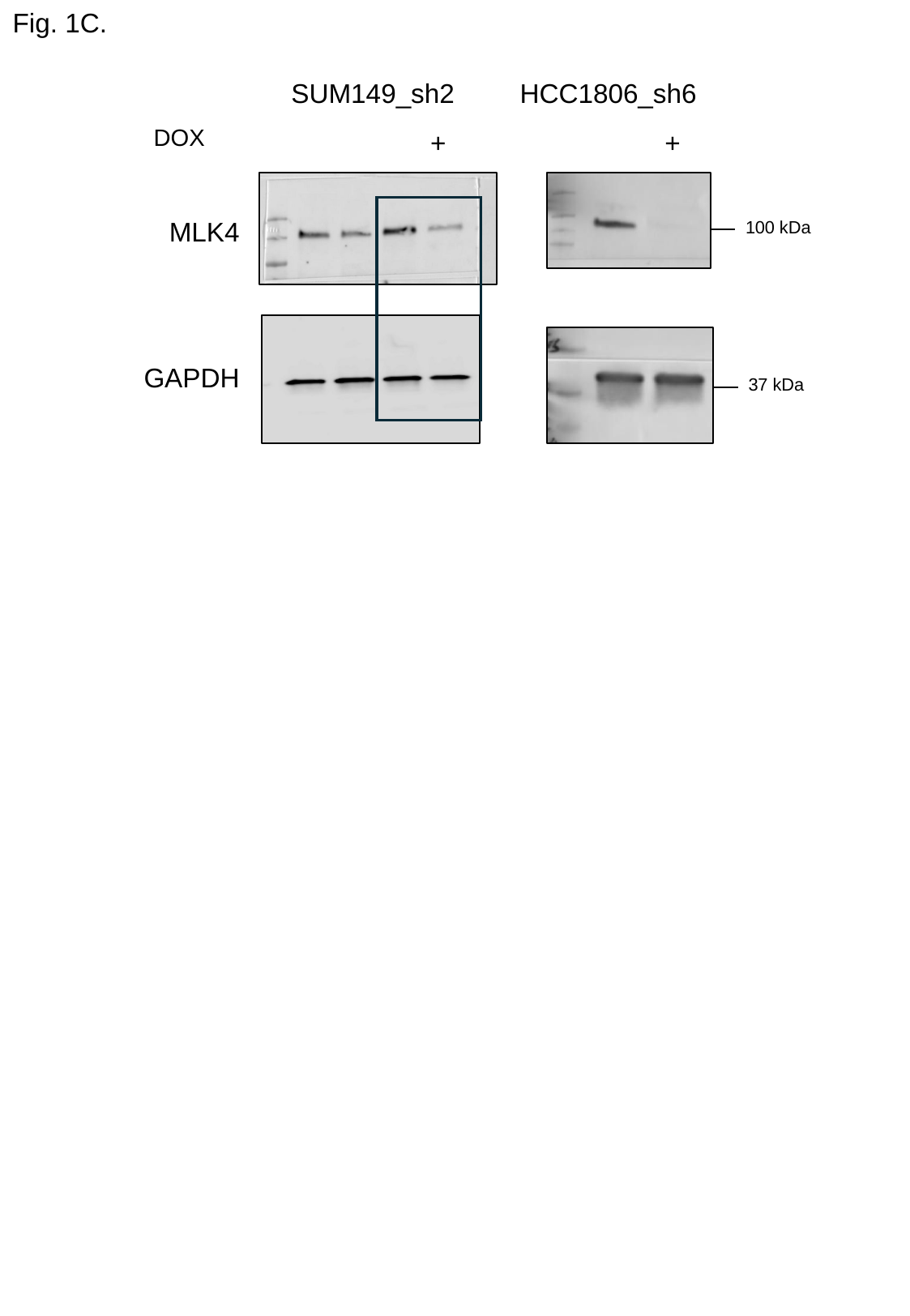

Fig. 1C.
SUM149_sh2
HCC1806_sh6
DOX
+
+
MLK4
GAPDH
100 kDa
37 kDa

## Slide 3
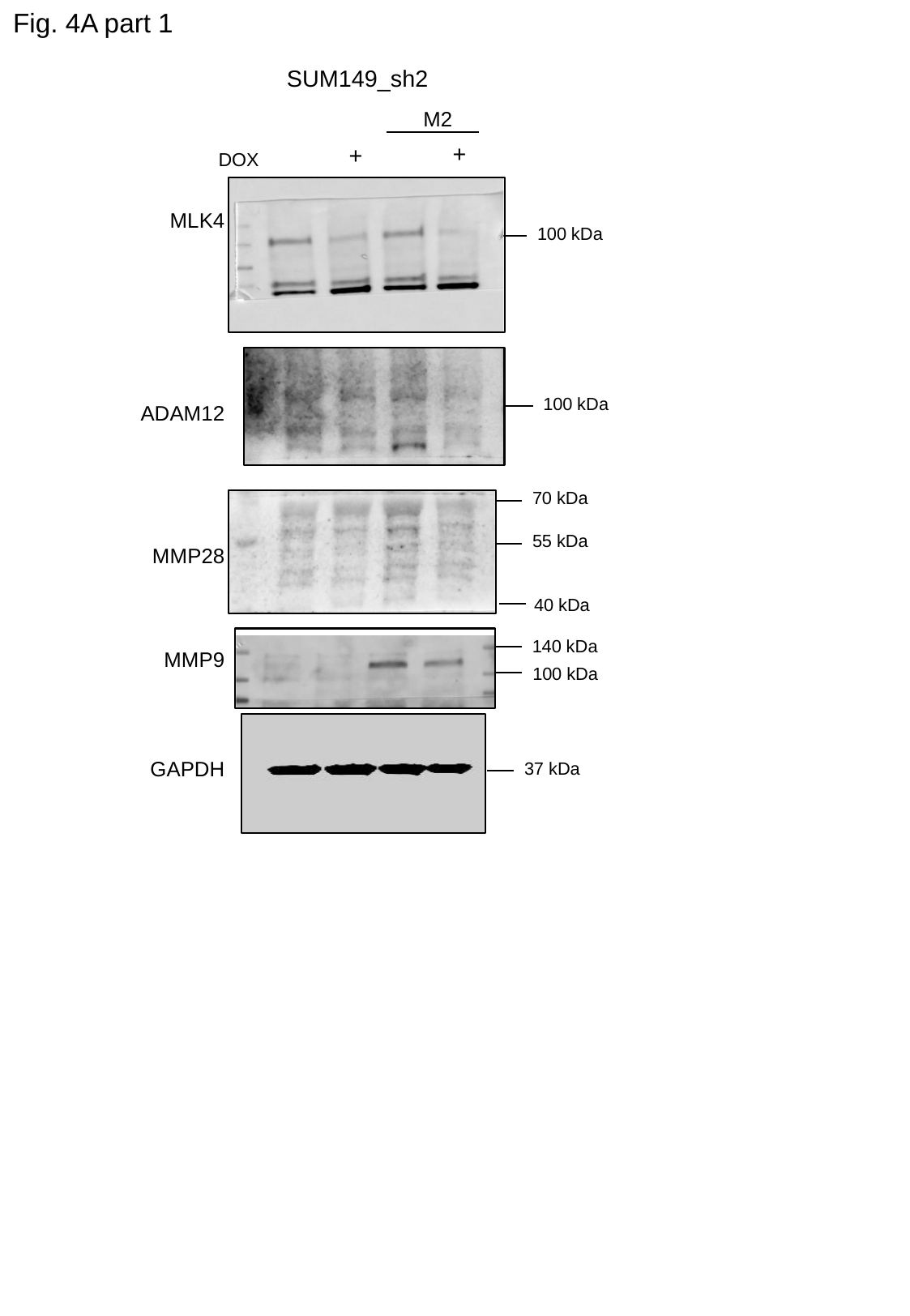

Fig. 4A part 1
SUM149_sh2
M2
+
+
DOX
MLK4
ADAM12
MMP28
MMP9
GAPDH
100 kDa
100 kDa
70 kDa
55 kDa
40 kDa
140 kDa
100 kDa
37 kDa

## Slide 4
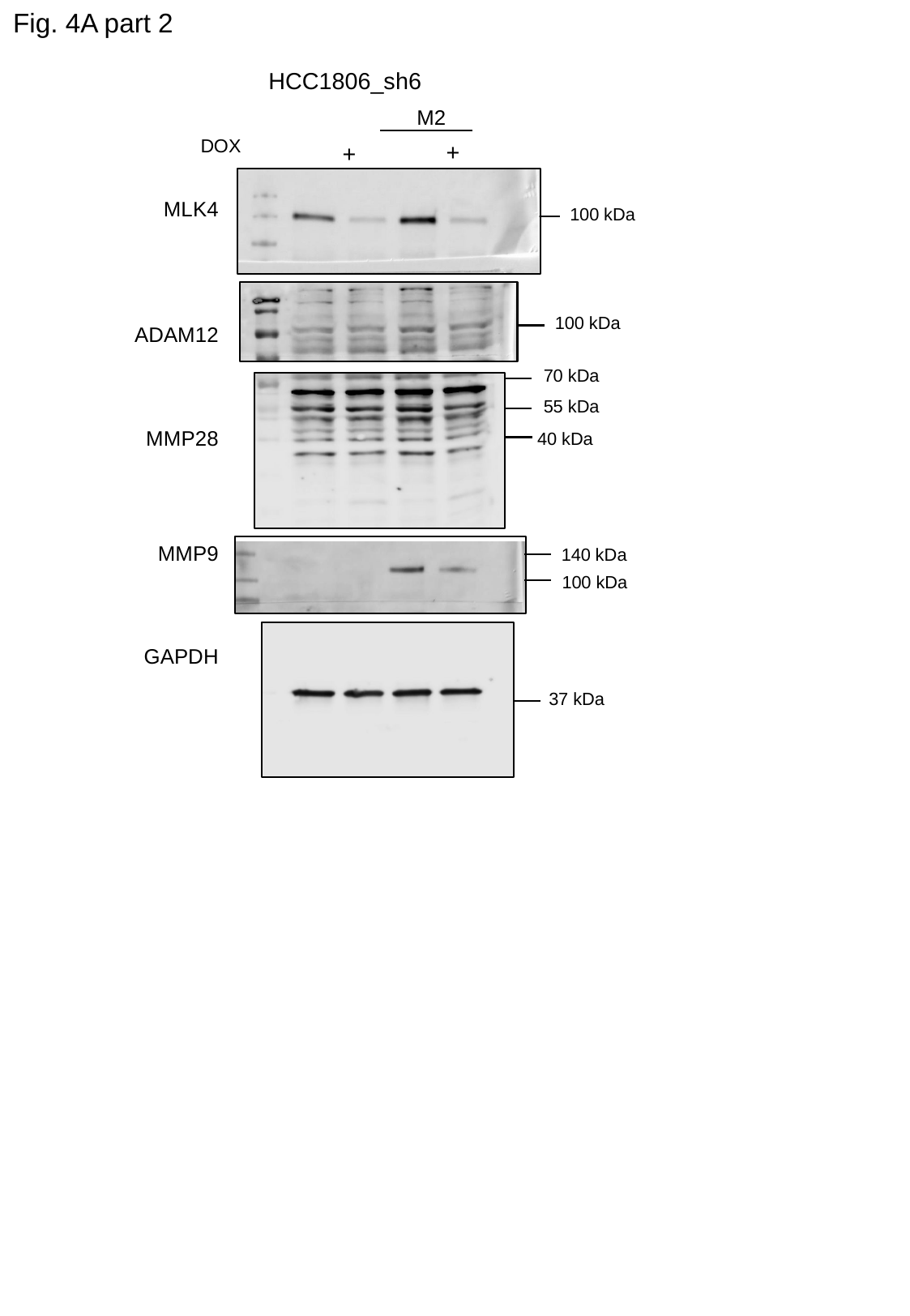

Fig. 4A part 2
HCC1806_sh6
M2
DOX
+
+
MLK4
ADAM12
MMP28
MMP9
GAPDH
100 kDa
100 kDa
70 kDa
55 kDa
40 kDa
140 kDa
100 kDa
37 kDa

## Slide 5
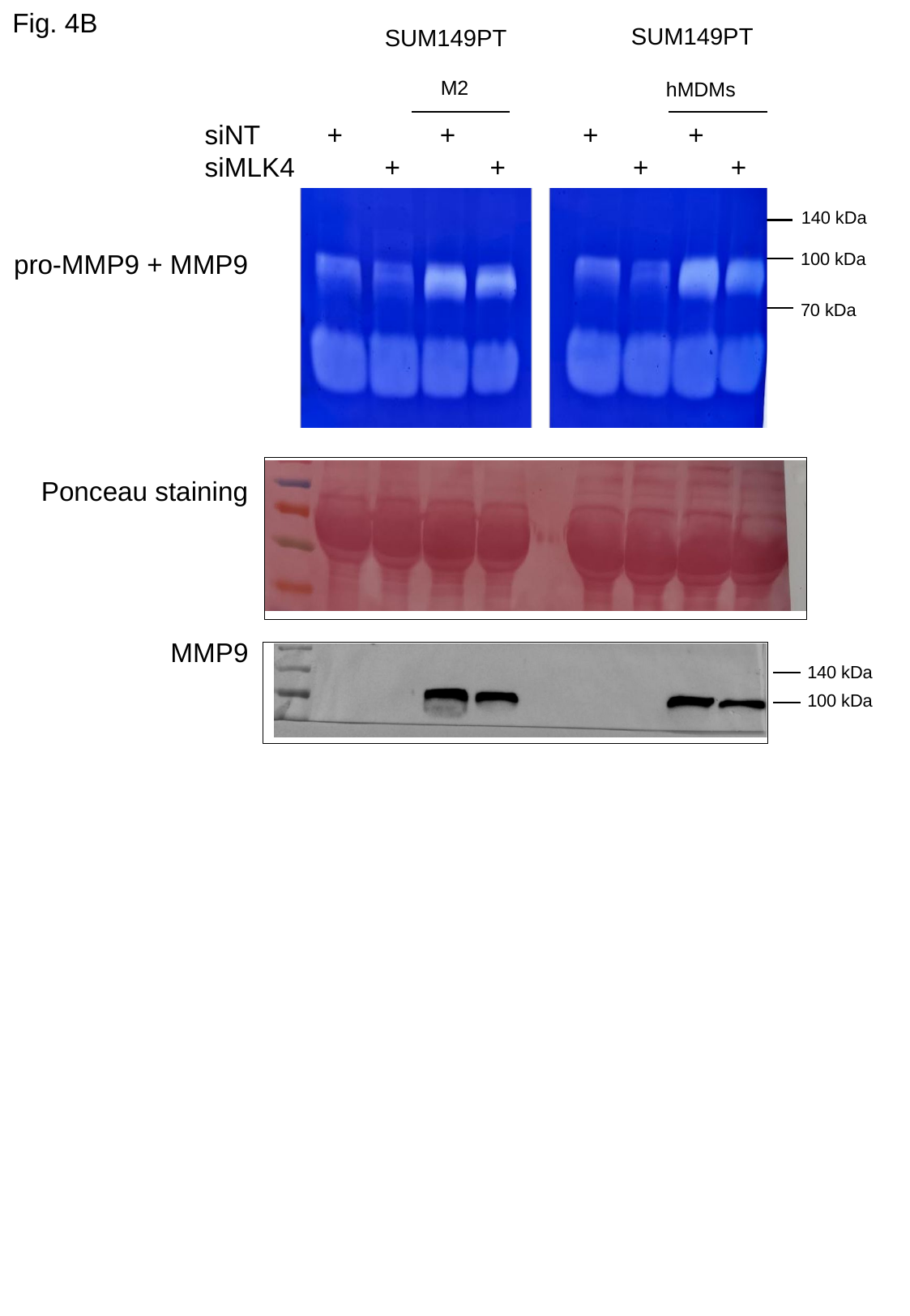

Fig. 4B
SUM149PT
SUM149PT
M2
hMDMs
siNT + + + +
siMLK4 + + + +
pro-MMP9 + MMP9
Ponceau staining
MMP9
140 kDa
100 kDa
70 kDa
1 0,6 1,9 1,2
1 0,7 2 1,3
140 kDa
100 kDa

## Slide 6
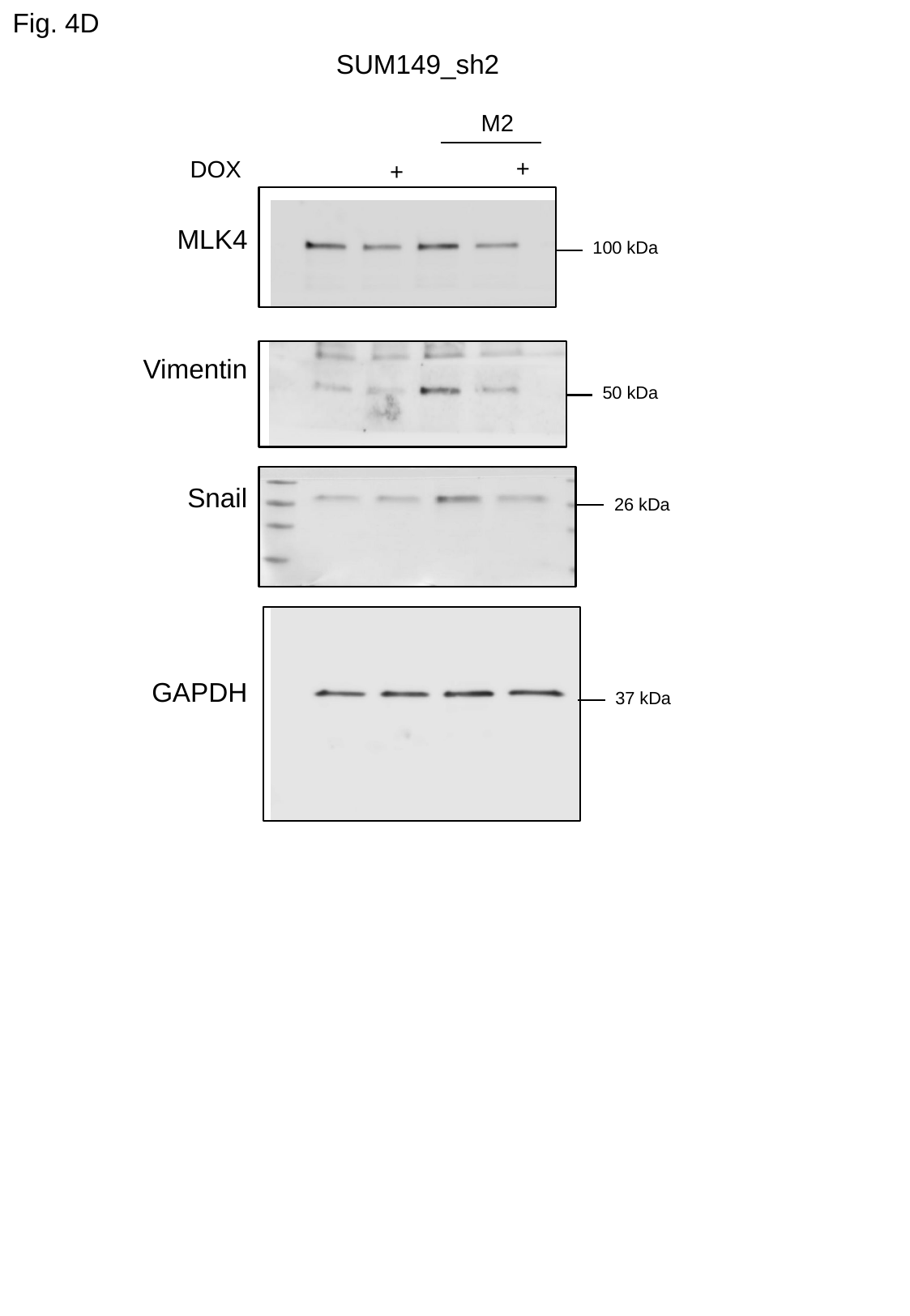

Fig. 4D
SUM149_sh2
M2
+
DOX
+
MLK4
Vimentin
Snail
GAPDH
100 kDa
50 kDa
26 kDa
37 kDa

## Slide 7
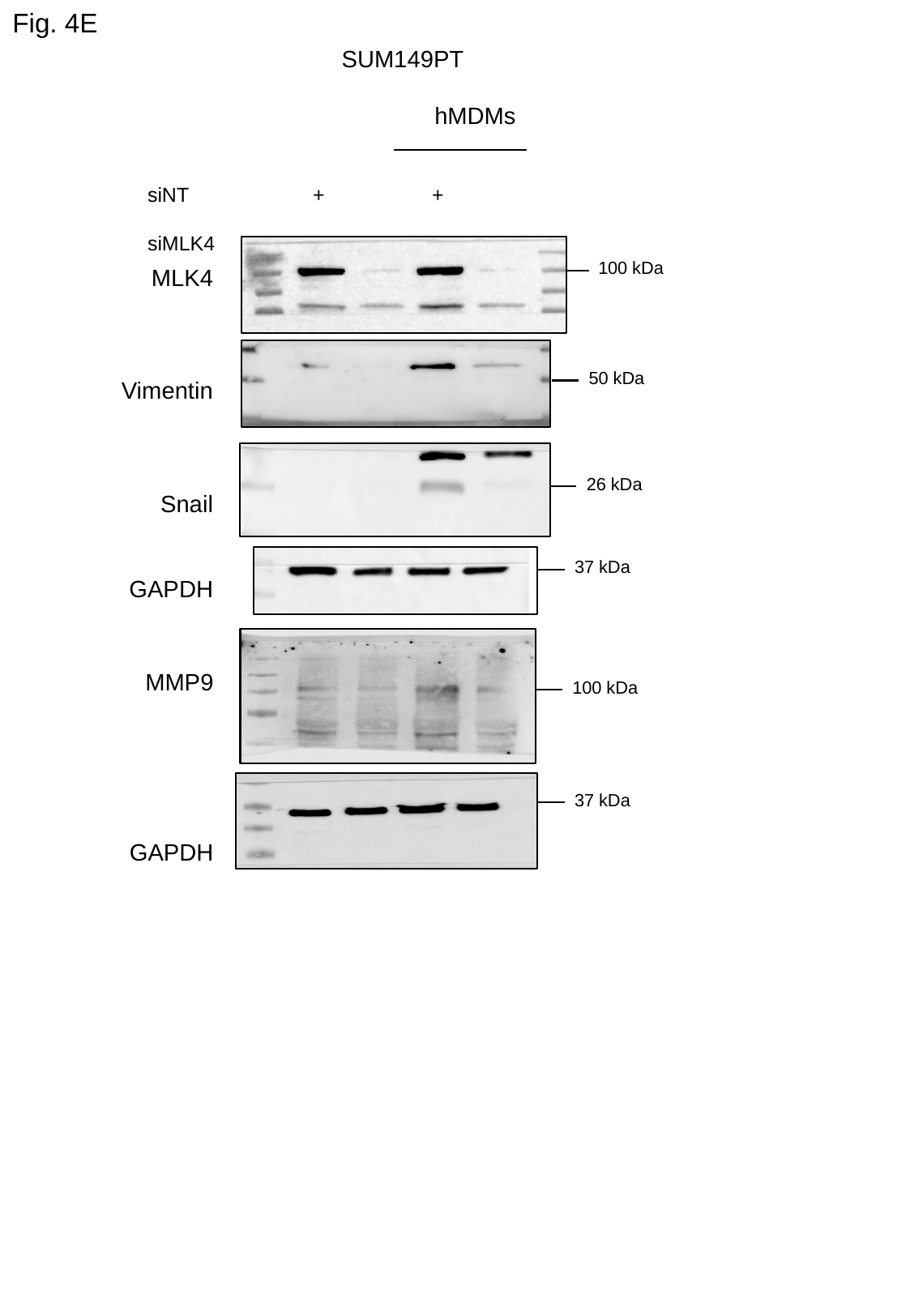

Fig. 4E
SUM149PT
hMDMs
siNT + +
siMLK4 + +
100 kDa
MLK4
Vimentin
Snail
GAPDH
50 kDa
26 kDa
37 kDa
MMP9
GAPDH
100 kDa
37 kDa

## Slide 8
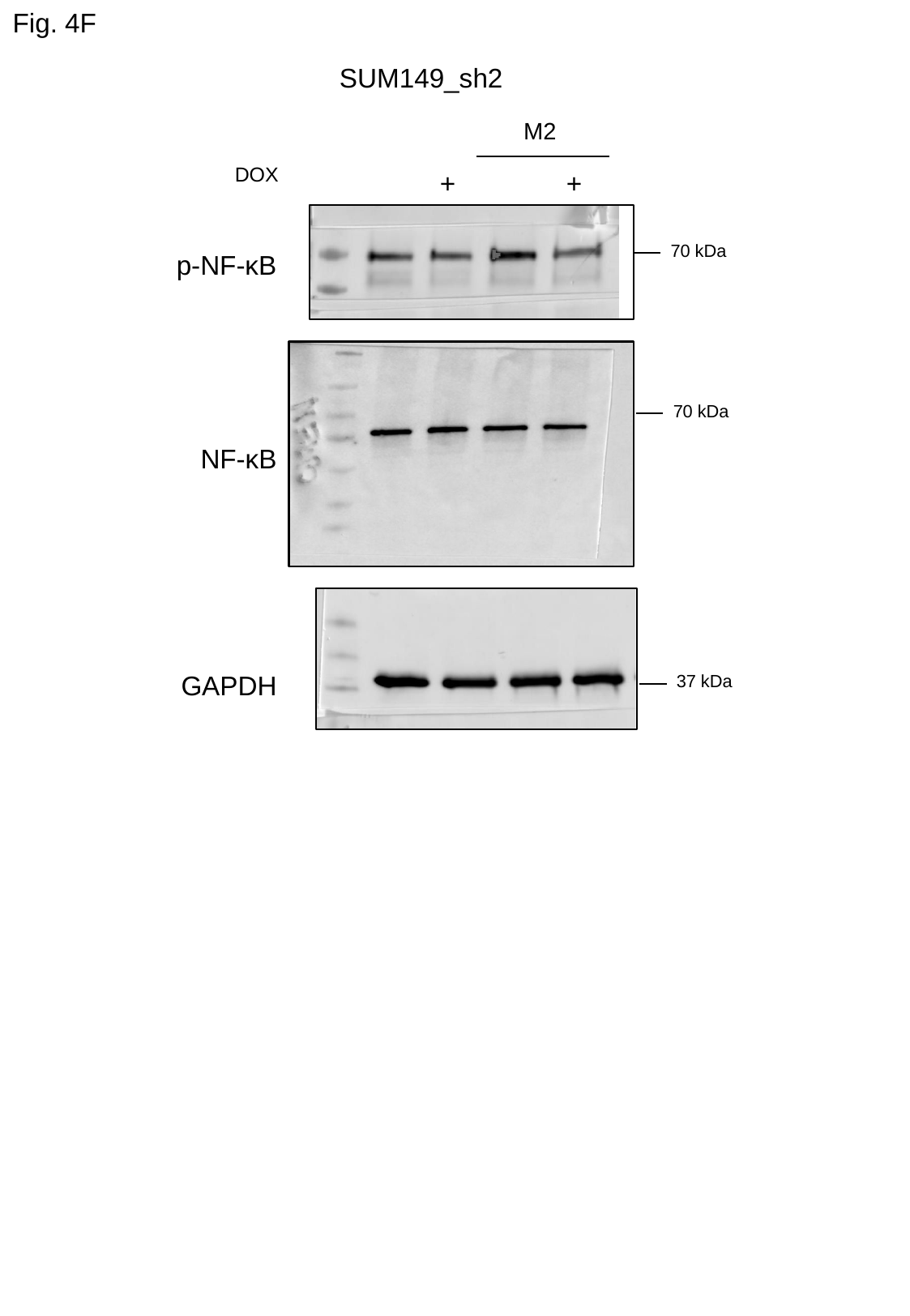

Fig. 4F
SUM149_sh2
M2
DOX
+
+
70 kDa
p-NF-κB
NF-κB
GAPDH
70 kDa
37 kDa

## Slide 9
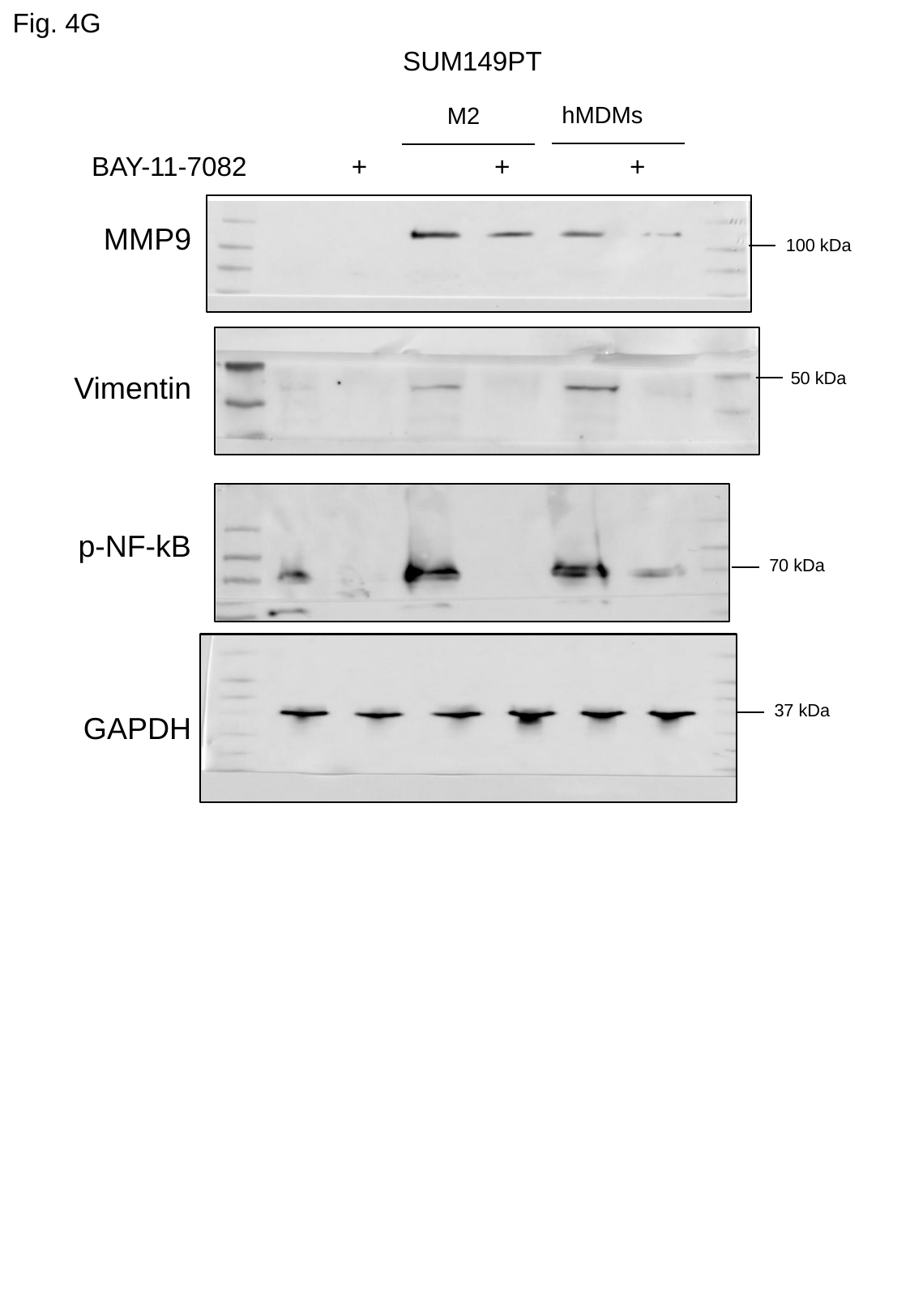

Fig. 4G
SUM149PT
hMDMs
M2
BAY-11-7082 + + +
MMP9
Vimentin
p-NF-kB
GAPDH
100 kDa
50 kDa
70 kDa
37 kDa

## Slide 10
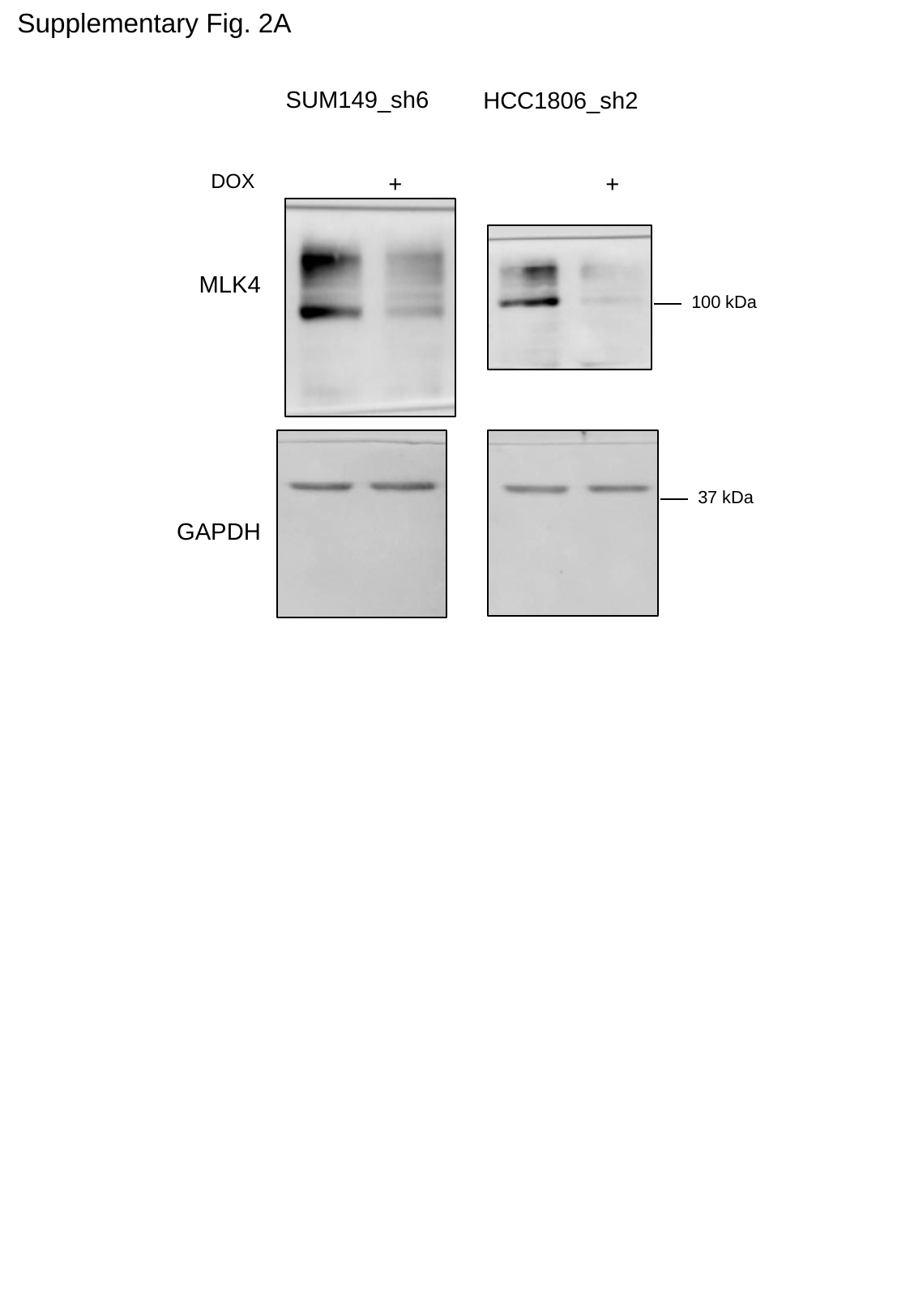

Supplementary Fig. 2A
SUM149_sh6
HCC1806_sh2
+
DOX
+
MLK4
GAPDH
100 kDa
37 kDa

## Slide 11
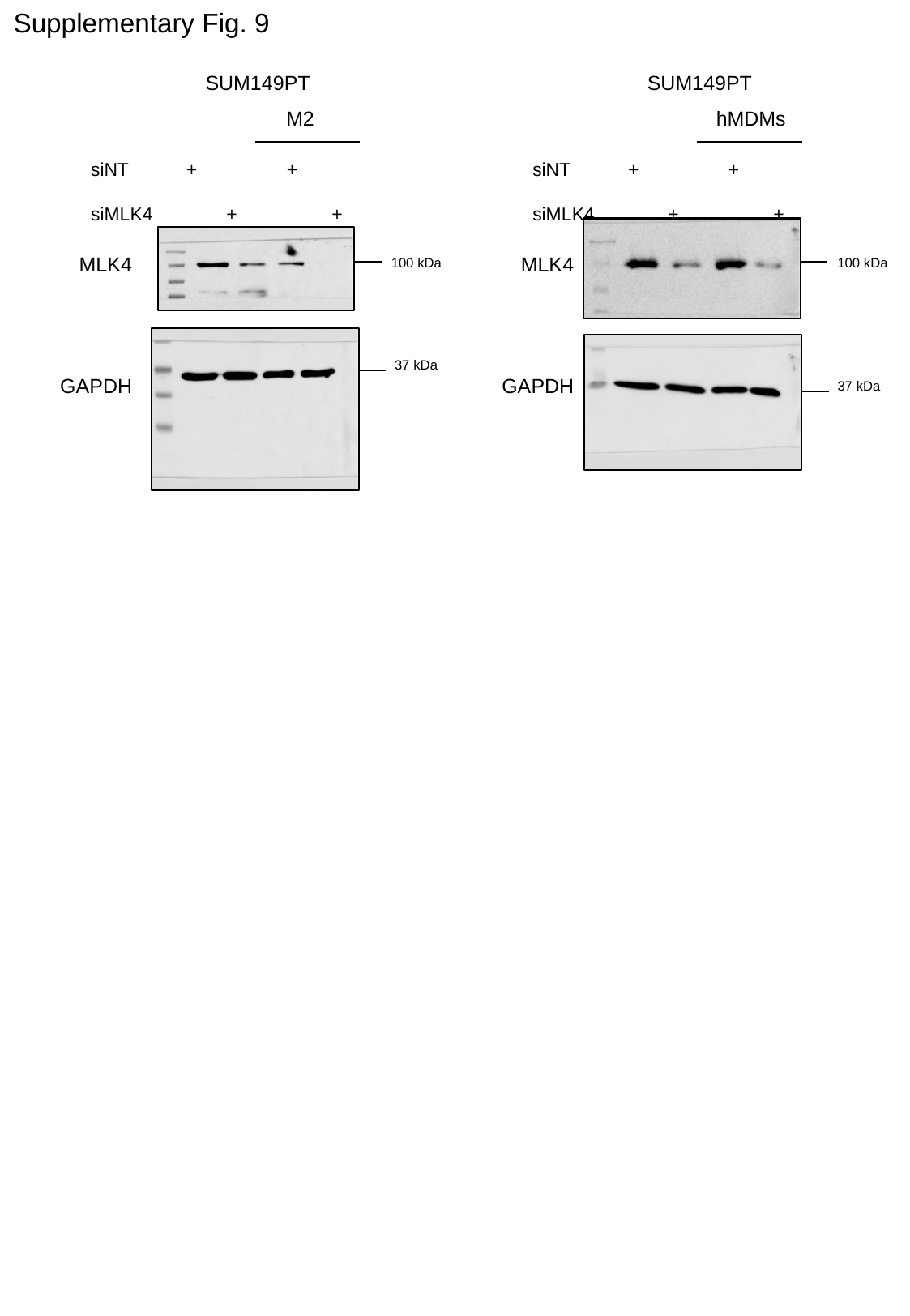

Supplementary Fig. 9
SUM149PT
SUM149PT
M2
hMDMs
siNT + +
siMLK4 + +
siNT + +
siMLK4 + +
MLK4
GAPDH
MLK4
GAPDH
100 kDa
100 kDa
37 kDa
37 kDa
